# Dynamic relationships among pathways producing hydrocarbons and fatty acids of maize silk cuticular waxes

**DOI:** 10.1093/plphys/kiae150

**Published:** 2024-03-27

**Authors:** Keting Chen, Liza E Alexander, Umnia Mahgoub, Yozo Okazaki, Yasuhiro Higashi, Ann M Perera, Lucas J Showman, Derek Loneman, Tesia S Dennison, Miriam Lopez, Reid Claussen, Layton Peddicord, Kazuki Saito, Nick Lauter, Karin S Dorman, Basil J Nikolau, Marna D Yandeau-Nelson

**Affiliations:** Department of Genetics, Development & Cell Biology, Iowa State University, Ames, IA 50011, USA; Bioinformatics & Computational Biology Graduate Program, Iowa State University, Ames, IA 50011, USA; Roy J. Carver Department of Biochemistry, Biophysics & Molecular Biology, Iowa State University, Ames, IA 50011, USA; Department of Genetics, Development & Cell Biology, Iowa State University, Ames, IA 50011, USA; Metabolomics Research Group, RIKEN Center for Sustainable Resource Science, Yokohama, Kanagawa 230-0045, Japan; Graduate School of Bioresources, Mie University, Tsu, Mie 514-8507, Japan; Metabolomics Research Group, RIKEN Center for Sustainable Resource Science, Yokohama, Kanagawa 230-0045, Japan; W.M. Keck Metabolomics Research Laboratory, Iowa State University, Ames, IA 50011, USA; W.M. Keck Metabolomics Research Laboratory, Iowa State University, Ames, IA 50011, USA; Department of Genetics, Development & Cell Biology, Iowa State University, Ames, IA 50011, USA; Department of Plant Pathology & Microbiology, Iowa State University, Ames, IA 50011, USA; Interdepartmental Genetics & Genomics Graduate Program, Iowa State University, Ames, IA 50011, USA; Corn Insects and Crop Genetics Research Unit, USDA-ARS, Ames, IA 50011, USA; Department of Genetics, Development & Cell Biology, Iowa State University, Ames, IA 50011, USA; Department of Plant Pathology & Microbiology, Iowa State University, Ames, IA 50011, USA; Interdepartmental Genetics & Genomics Graduate Program, Iowa State University, Ames, IA 50011, USA; Metabolomics Research Group, RIKEN Center for Sustainable Resource Science, Yokohama, Kanagawa 230-0045, Japan; Department of Plant Pathology & Microbiology, Iowa State University, Ames, IA 50011, USA; Interdepartmental Genetics & Genomics Graduate Program, Iowa State University, Ames, IA 50011, USA; Corn Insects and Crop Genetics Research Unit, USDA-ARS, Ames, IA 50011, USA; Department of Genetics, Development & Cell Biology, Iowa State University, Ames, IA 50011, USA; Bioinformatics & Computational Biology Graduate Program, Iowa State University, Ames, IA 50011, USA; Department of Statistics, Iowa State University, Ames, IA 50011, USA; Bioinformatics & Computational Biology Graduate Program, Iowa State University, Ames, IA 50011, USA; Roy J. Carver Department of Biochemistry, Biophysics & Molecular Biology, Iowa State University, Ames, IA 50011, USA; Interdepartmental Genetics & Genomics Graduate Program, Iowa State University, Ames, IA 50011, USA; Center for Metabolic Biology, Iowa State University, Ames, IA 50011, USA; Department of Genetics, Development & Cell Biology, Iowa State University, Ames, IA 50011, USA; Bioinformatics & Computational Biology Graduate Program, Iowa State University, Ames, IA 50011, USA; Interdepartmental Genetics & Genomics Graduate Program, Iowa State University, Ames, IA 50011, USA; Center for Metabolic Biology, Iowa State University, Ames, IA 50011, USA

## Abstract

The hydrophobic cuticle is the first line of defense between aerial portions of plants and the external environment. On maize (*Zea mays* L.) silks, the cuticular cutin matrix is infused with cuticular waxes, consisting of a homologous series of very long-chain fatty acids (VLCFAs), aldehydes, and hydrocarbons. Together with VLC fatty-acyl-CoAs (VLCFA-CoAs), these metabolites serve as precursors, intermediates, and end-products of the cuticular wax biosynthetic pathway. To deconvolute the potentially confounding impacts of the change in silk microenvironment and silk development on this pathway, we profiled cuticular waxes on the silks of the inbreds B73 and Mo17, and their reciprocal hybrids. Multivariate interrogation of these metabolite abundance data demonstrates that VLCFA-CoAs and total free VLCFAs are positively correlated with the cuticular wax metabolome, and this metabolome is primarily affected by changes in the silk microenvironment and plant genotype. Moreover, the genotype effect on the pathway explains the increased accumulation of cuticular hydrocarbons with a concomitant reduction in cuticular VLCFA accumulation on B73 silks, suggesting that the conversion of VLCFA-CoAs to hydrocarbons is more effective in B73 than Mo17. Statistical modeling of the ratios between cuticular hydrocarbons and cuticular VLCFAs reveals a significant role of precursor chain length in determining this ratio. This study establishes the complexity of the product–precursor relationships within the silk cuticular wax-producing network by dissecting both the impact of genotype and the allocation of VLCFA-CoA precursors to different biological processes and demonstrates that longer chain VLCFA-CoAs are preferentially utilized for hydrocarbon biosynthesis.

## Teaser

Precursor chain length, plant genotype, and spatio-temporal dynamic gradients impact product–precursor relationships in the cuticular wax-producing network of maize silks.

## Introduction

The cuticle is the external hydrophobic barrier covering the epidermis of most aerial organs of terrestrial plants (Riederer and Schreiber 2001). The cuticle limits transpirational water loss (Yeats and Rose 2013) and thus has a role in protecting the organism from such abiotic stresses as drought, salinity, and temperature. Additional protective roles include protection from ultraviolet radiation (Krauss et al. 1997; Shepherd and Wynne Griffiths 2006), and from biotic stresses, such as fungal and bacterial pathogens and herbivory by insects (Eigenbrode and Espelie 1995; Serrano et al. 2014).

The cuticle is comprised of a cutin polyester matrix that is infused with and laid atop by solvent-extractable cuticular waxes, which themselves have been shown to be associated with protection against ultraviolet radiation (Tafolla-Arellano et al. 2018), drought (Panikashvili et al. 2007; Kosma et al. 2009), humidity changes (Kim et al. 2019), and disease resistance (Wang et al. 2020). In general, these extracellular cuticular waxes can include acyl derivatives such as very long-chain fatty acids (VLCFAs) (i.e. fatty acids of 20-carbons and longer), hydrocarbons, aldehydes, primary and secondary alcohols, ketones, and wax esters. Additional components include triterpene derivatives, such as β-sitosterol, stigmasterol, lupeol, and α- and β-amyrins (Jetter et al. 2006). The specific composition of the cuticular wax metabolome is dependent on the organism, tissue or organ, and temporal stage of tissue or organ development (Shepherd and Wynne Griffiths 2006; Samuels et al. 2008). For example, in maize (*Zea mays* L.), the cuticular waxes on juvenile and adult leaves are primarily composed of VLCFAs, alcohols, aldehydes, and wax esters, with only about 1% and 17% being hydrocarbons, respectively (Bianchi et al. 1984, 1985). In contrast, on maize silks, which are the stigmatic floral tissues that receive pollen and facilitate fertilization of the ovule, cuticular waxes are particularly rich in hydrocarbons, comprising 40% to 90% of these lipids, with lesser amounts of VLCFAs, and minor to trace amounts of aldehydes and alcohols (Yang et al. 1992; Perera et al. 2010; Loneman et al. 2017; Dennison et al. 2019). Hence, the metabolic network responsible for cuticular wax deposition on silks include two major branches of the pathway, one that produces hydrocarbons and one that produces VLCFAs.

The favored model for hydrocarbon biosynthesis in plants is that it occurs in the endoplasmic reticulum via the reduction of a saturated or unsaturated VLC fatty acyl-CoA (VLCFA-CoA) to an aldehyde intermediate, which subsequently undergoes decarbonylation to produce either a saturated (alkane) or unsaturated (alkene) hydrocarbon, which are then transported to the extracellular cuticle (Cheesbrough and Kolattukudy 1984; Jetter et al. 2006; Bernard et al. 2012). Because the majority of VLCFA-CoAs are comprised of an even number of carbon atoms (*2n*, where *n* represents the number of 2-carbon units used to assemble the VLCFA-CoA), this process generates hydrocarbon products with an odd-numbered carbon chain length (i.e. *2n − 1*). However, if the process begins with odd-numbered VLCFA-CoA (i.e. *2n + 1* carbons) substrates, even-numbered hydrocarbon products are produced (i.e. (*2n + 1*) *− 1 = 2n*). Such even-numbered hydrocarbons occur at lower quantities in many cuticles, including that of maize silks (Yang et al. 1992; Loneman et al. 2017; Dennison et al. 2019). The sequence of hydrocarbon-forming reactions can occur at each VLCFA-CoA chain length (i.e. at every value of *2n* or *2n + 1*), thereby generating a homologous series of cuticular hydrocarbon products with alkyl chain lengths ranging from 19 to 33 carbon atoms (Perera et al. 2010). Apart from allocation to the reduction–decarbonylation pathway, VLCFA-CoA precursors of every chain length can also be hydrolyzed into free VLCFAs and subsequently transported to the cuticle as cuticular free VLCFAs. Importantly, the cuticular VLCFAs, the aldehyde intermediates and the hydrocarbon products of the reduction–decarbonylation pathway are all solvent-extractable from silk cuticles (Loneman et al. 2017), permitting the assessment of relationships among these metabolically interconnected lipids. Indeed, based on the assessment of cuticular VLCFAs, aldehydes, alkanes, and alkenes collected from maize silks, a pathway model involving a series of parallel reactions has been proposed for the biosynthesis of both alkanes and alkenes (Perera et al. 2010; Gonzales-Vigil et al. 2017).

As with all biological processes, this cuticular wax-producing network is genetically programmed and is regulated by many factors that integrate environmental and developmental cues. Indeed, both genetic and environmental factors impact the cuticular wax metabolome on maize silks. For example, multiple characterizations of the maize silk cuticular waxes revealed substantial difference (i.e. up to 10-fold) in abundance among various panels of inbred lines, demonstrating the breadth of natural variation in cuticular composition (Yang et al. 1992; Miller et al. 2003; Perera et al. 2010; Loneman et al. 2017; Dennison et al. 2019). Moreover, within any individual maize inbred line, cuticular wax accumulation varies along the length of the silks. Specifically, cuticular hydrocarbons accumulate at up to 5-fold higher levels on the portions of silks that have emerged from the protective husk leaves, as compared to the portions that are still encased by these husk leaves (Miller et al. 2003; Perera et al. 2010; Loneman et al. 2017; Dennison et al. 2019), demonstrating an impact of silk microenvironment on hydrocarbon biosynthesis. Thus, it is precisely the dynamic character of these natural systems that permits the statistical interrogation of the hydrocarbon-producing network among maize inbreds.

Herein, a fine dissection of the cuticular wax-producing network is conducted via cuticular wax profiling of five contiguous silk sections, from base to tip, for the maize inbred lines B73 and Mo17, as well as their reciprocal hybrids. These silk sections constitute a spatio-temporal gradient that captures the spatial differences in cuticular wax composition and differences caused by silk development within the encased portions of the silks (Fuad-Hassan et al. 2008). B73 and Mo17 were selected for these studies because they are two agronomically important inbred lines for which high quality genome sequences and annotations are available (Hufford et al. 2021; Chen et al. 2023). Moreover, previous studies have revealed wide variation in cuticular wax composition between these two inbreds, which was the basis for deducing the potential pathway for alkene biosynthesis (Perera et al. 2010). Additionally, these inbred lines provide the genetic foundation for commonly used intermated B73 × Mo17 recombinant inbred line populations that have facilitated the quantitative genetic analysis of metabolite traits (González-Rodríguez et al. 2023), including cuticular waxes (Dennison 2020). Supervised and unsupervised multivariate statistical analyses of the cuticular wax profiles along the silk length of these maize lines reveal that both microenvironment and genotype of the silks greatly impact cuticular wax compositional dynamics. Furthermore, dissection of the product–precursor relationship within the silk cuticular wax-producing network demonstrated that with increasing acyl chain lengths, VLCFA-CoA precursors were preferentially incorporated into the hydrocarbon-producing pathway among many other processes that also utilize VLCFA-CoAs (e.g. complex lipid biosynthesis). These findings provide the foundation for unraveling the complex metabolic networks underlying the compositional variations that are observed in cuticular waxes in response to changes in micro-environment and across different genetic backgrounds.

## Results

**Figure 1.**
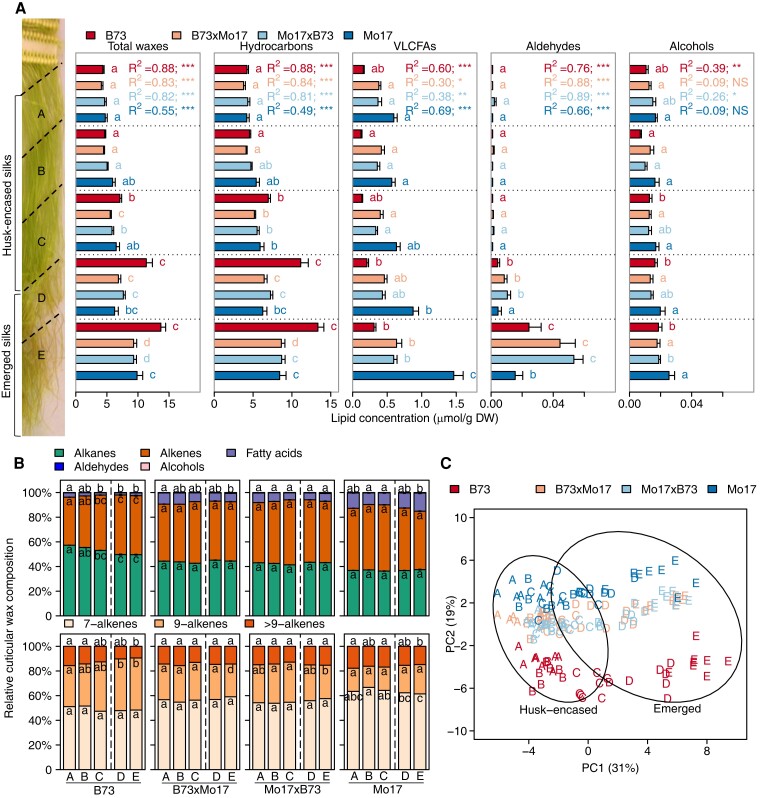
Spatio-temporal cuticular wax profiles of maize silks from inbreds Mo17 and B73, and their reciprocal hybrids. **A)** Concentrations of total cuticular waxes and individual lipid classes from silks harvested 3 days postsilk emergence and cut into five allometric sections, A–E (see Materials and Methods). The bold dashed line depicts the transition point between husk-encased (sections A, B, and C) and emerged (sections D and E) portions of the silks. For each genotype, the changes in concentration of each cuticular wax class along the A to E gradient were fitted to a quadratic regression model. The resulting *R*^2^ values are listed and the associated *P*-values are denoted by asterisks (***, *P* < 0.0001; **, *P* < 0.001; *, *P* < 0.05; NS, nonsignificant). Different letters associated with data bars of the same color denote a statistically significant difference in accumulation between silk sections within a genotype (*P* < 0.05; Tukey's Honestly Significant Difference (HSD) test). Seven to eight biological replicates (two ears pooled from two separate plants per replicate) were evaluated per combination of genotype and silk section, constituting a total sample size of 158. **B)** Proportion of each cuticular wax class relative to total cuticular wax accumulation. Aldehydes and alcohols comprise <1% of the cuticular wax metabolome and are not depicted in the figure. Alkenes are comprised predominantly of 7- and 9-monoene classes, and “>9” constituents that include 14- and 15-monoenes, and two dienes. Different letters within data bars for a given genotype denote a statistically significant difference in cuticular wax relative abundance among silk sections for a given metabolite class (*P* < 0.05; Tukey's HSD test). **C)** Principal component analysis (PCA) of cuticular wax metabolite abundances among different silk sections. Each data point, which represents the concentrations of the 45 cuticular wax metabolites profiled from each silk sample, is labeled by silk section (A–E) and color-coded according to genotype. Percentages represent the amounts of variance explained by the first and second principal components (PC1 and PC2). Ovals represent 95% confidence ellipses for emerged and husk-encased samples. Abbreviations: DW, dry weight; HSD, honestly significant difference.

To model the metabolic network for hydrocarbon production and deconvolute the potential impacts of development, microenvironment, and genetics on the dynamics of the cuticular wax metabolome, we profiled extracellular cuticular waxes along the lengths of silks collected at 3 days postsilk emergence. These silks were exhaustively sampled (∼20 biological replicates) from four maize genotypes (inbred lines B73 and Mo17, and their reciprocal hybrids, B73×Mo17 and Mo17×B73) in the summer growing seasons of 2014 and 2015. The collected silks were dissected into five contiguous segments, named sections A–E, from base to tip (Fig. 1A). At the time of silk collection, cell division has ceased, and the developmental gradient is primarily determined by cellular elongation, which occurs only in the husk-encased portions of the silk, with an acropetal decrease in the rate of cell elongation (Fuad-Hassan et al. 2008). Therefore, these sections reflect a combination of spatial and temporal gradients (Fuad-Hassan et al. 2008). This gradient includes the microenvironment transition of the silks, as sections A–C were husk-encased, and sections D and E had emerged into the external environment (McNinch et al. 2020). These factors were analyzed with the 2014 dataset, and the comparison to the 2015 data allowed for the analysis of macroenvironmental effects. All cuticular wax composition data are available in Supplementary Table S1.

### Spatio-temporal dynamic changes in the cuticular wax metabolome in maize silks of different genotypes

Depending on genotype and silk section, hydrocarbons account for 85% to 97%, and VLCFAs account for 2% to 15%, of total extractable cuticular waxes; whereas aldehydes and alcohols are detected only in trace amounts (<1%) (Fig. 1, A and B; Supplementary Table S1). Irrespective of the genotype, total cuticular wax load increases acropetally (i.e. increases from sections A to E; Fig. 1A). A 3-fold increase in total cuticular wax abundance is observed along the silk length for inbred line B73, whereas total abundance only doubles for inbred line Mo17 and the two reciprocal hybrids (Fig. 1A). Furthermore, accumulation of these lipids is nonlinear, and changes occur most abruptly at the transition between encased and emerged portions of silks (i.e. sections C and D; Fig. 1A), and along the emerged portions of silks (i.e. sections D and E; Fig. 1A). Regression of the cuticular wax concentration along the silk length reveals that a quadratic polynomial model (adjusted *R*^2^ ranging from 0.2 to 0.9), as compared to a linear model (Supplementary Table S2), better depicts the dynamics of total waxes, hydrocarbons, VLCFAs, and aldehydes, underscoring the complexity that underlies the dynamics of cuticular wax accumulation.

The compositional dynamics of these chemical classes vary not only along the silk length but also among the different genotypes. Silks from inbred B73 and hybrid B73×Mo17 exhibited a decrease in the relative abundance of VLCFAs and a concomitant increase in relative abundance of hydrocarbons along the silk length (Fig. 1B). In contrast, there was no change in the relative abundance of VLCFAs along this spatio-temporal gradient in the silks of Mo17×B73, and there was no consistent change for Mo17 (Fig. 1B). However, across this gradient, cuticular VLCFA concentrations were 1.7- to 4.7-fold higher in Mo17 and both reciprocal hybrids, as compared to the B73 inbred. The largest difference among the genotypes occurs for emerged silks (sections D and E) (Fig. 1A), suggesting that in Mo17 VLCFA-CoA precursors are preferentially converted to VLCFAs and transported to the silk surface instead of first being utilized for hydrocarbon production and then transported to the silk surface.

Hydrocarbon composition, specifically the degree of unsaturation (i.e. alkenes), also varies among these genotypes. Although alkenes consistently comprise ∼50% of the hydrocarbons along the silk length of Mo17 and the reciprocal hybrids, for B73 the relative abundance of alkenes increased from approximately 40% at the base to 50% of hydrocarbons at the tips of the silks (Fig. 1B). Most alkenes harbor a single double bond (i.e. a monoene), which is positioned either at the 7th or 9th carbon-position, irrespective of the alkyl chain-length (Fig. 1B). However, additional, previously characterized (Perera et al. 2010; Dennison et al. 2019), minor monoenes were also identified with chain lengths of 29, 31, and 33 carbon chain lengths and double bonds positioned at the 14th or 15th carbon positions. Accumulation patterns of the 7- and 9-monoenes differed between the two inbred lines (Fig. 1B). Specifically, for B73, 7-monoenes consistently comprised ∼50% of all alkenes along the silk length, while the relative abundance of 9-monoenes increased from 33% to 42% (Fig. 1B) of the alkenes from base to tip of the silks. In contrast, on Mo17 silks, the relative abundance of both the 7- and 9-monoenes did not change along the silk length, and they accounted for 64% and 20% of all alkenes, respectively (Fig. 1B). Thus, although the absolute amounts (µmol/g dry weight tissue) of these alkenes increased by ∼2.5-fold along the silk length (Supplementary Fig. S1), the relative proportions of 7- and 9-monenes remained constant for Mo17, with the 9-monoenes being less abundant (Fig. 1B).

The relative impacts of genotype, silk development, and silk encasement status (i.e. silk microenvironment) on the dynamics of the cuticular wax metabolome were visualized via principal component analysis (PCA) of the 45 cuticular wax metabolites (Fig. 1C). The distribution of silk samples along PC1, which accounts for 31% of the total variance, is tightly associated with the position along the silk length, and mainly separates the husk-encased (sections A–C) from the emerged (sections D and E) portions of the silks. Moreover, PC1 also separates samples based upon silk development, separating section C from sections A and B within the husk-encased silks of the inbred B73 and the two hybrids, demonstrating the impact of the temporal gradient caused by cell elongation in the encased portions of the silks (Fuad-Hassan et al. 2008). PC2 accounts for 19% of the total variance, and primarily separates samples according to genotype, with inbred lines B73 and Mo17 forming subclusters, and the hybrids forming a third cluster, which is closer to Mo17 than B73 (Fig. 1C).

Hence, we hypothesize that the spatio-temporal gradient of the silk has a greater influence on cuticular wax accumulation than the genetic variation that was evaluated in this study. Two-way ANOVA on specific wax classes (i.e. hydrocarbon, aldehyde, and alcohol) and each individual metabolite (Supplementary Tables S3 and S4) supports this hypothesis and reveals that the position along the length of the silk is the major determinant of the observed variance in total cuticular wax accumulation (accounting for 70% of the variance). The effect of genotype and the two-way interaction between genotype and silk section explain a much smaller proportion (between 5% and 10%) of the variance in the accumulation of these components (Supplementary Table S4). In stark contrast, but in agreement with the major patterns presented in Fig. 1B, the majority of the observed variance in cuticular VLCFA accumulation is explained primarily by genotype (66%), whereas the position along the length of the silk explains only 17% of this variation.

### Silk microenvironment is the major driver of changes in cuticular wax accumulation

The variation in the cuticular waxes along the silk length may be explained by either or both the silk developmental gradient and/or the change in the microenvironment of the silks associated with husk-encasement status. The potentially confounding determinants of silk cellular development and silk encasement status were untangled by the application of a nested ANOVA model. Silk encasement status explained 57% of the variation in total cuticular wax abundance, whereas the silk acropetal gradient (i.e. sections A versus B versus C for encased portions, and section D versus E for emerged portions) explained only 13% of the observed variance (Supplementary Table S4). One can conclude therefore, that cuticular wax accumulation, at 3 days postsilk emergence, is primarily impacted by the change in silk microenvironment, associated with emergence of the silks from the protective husk leaves.

### Correlations among cuticular wax metabolites along the silk length reveal genotype-dependent clustering patterns

Here, we sought to evaluate the co-expressed metabolite networks and compare these networks among different genotypes. Spearman correlation matrices were calculated between every two detected wax metabolites along the silk length and these were used for subsequent weighted gene correlation network analysis (WGCNA) (Langfelder and Horvath 2008). WGCNA is a method that was first developed for identifying clusters of co-expressed genes but is also widely applied to metabolomics data (Shen et al. 2013; Bartzis et al. 2017; Rosato et al. 2018).

**Figure 2.**
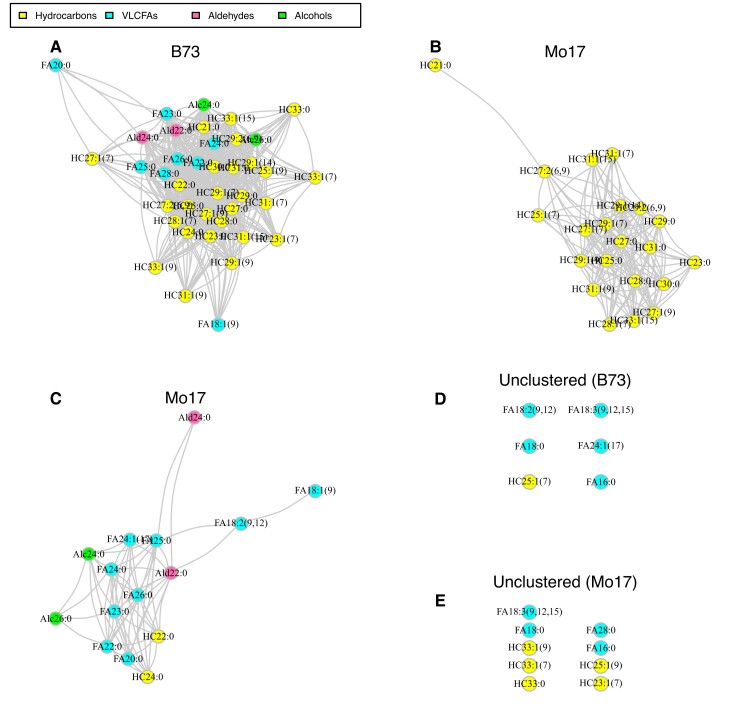
Correlation-based clustering of silk cuticular wax abundance data for inbred lines, B73 and Mo17. Rank-based Spearman correlations were calculated between all pairs of metabolites and used to construct the weighted correlation networks via weight gene correlation network analysis for **A)** B73 and **B** and **C)** Mo17. The nonclustered metabolites for B73 and Mo17 are shown in **D** and **E)** respectively. Pairs of lipid metabolites connected by edges are significantly correlated with correlation coefficients ≥0.5 and reside within the same cluster. Edge length represents correlation strength with shorter edges representing stronger correlations between metabolites. Nonclustered singleton metabolites were not statistically correlated with any other metabolites, or shared correlation values <0.5. Abbreviations: Alc, alcohol; Ald, aldehyde; FA, fatty acid; HC, hydrocarbon; VLCFA, very long-chain fatty acid.

The cluster membership of many metabolites differs among the four genotypes that were evaluated (Fig. 2; Supplementary Fig. S2). For the inbred B73, and the reciprocal hybrids, the majority of metabolites group into a single cluster (Table 1, Fig. 2A; Supplementary Fig. S2) that includes hydrocarbons (*HC_2n-1_* and *HC_2n_*) and VLCFAs. In contrast, for the inbred Mo17, hydrocarbons, and VLCFAs mostly reside in two separate clusters (Table 1; Fig. 2, B and C). Frobenius vector norms were calculated from the Spearman correlation matrices to provide aggregate measures of the level of correlation among all metabolite pairs as they are modulated by the spatio-temporal silk gradient of a given genotype, as detailed in the Supplementary Methods. The Frobenius norms for silks of B73 and the reciprocal hybrids were statistically higher than for silks of Mo17 (Table 1), demonstrating that there are stronger correlations among the profiled metabolites in each of the former genotypes than Mo17. Collectively, these observed differences in correlation network topologies suggest that the dynamics of cuticular wax accumulation differ between Mo17 and B73, especially the dynamics of VLCFA accumulation.

**Table 1.** Silk cuticular wax metabolites from B73 and Mo17 exhibit different correlation patterns along the spatio-temporal silk gradient

Genotype	Frobenius norm	Number of clusters	Number of singletons
B73	0.58^A^	1	6
Mo17	0.48^B^	2	9
B73×Mo17	0.55^AB^	2	9
Mo17×B73	0.54^AB^	1	9

The Frobenius norm is derived from the sum of squares for all the elements in a Spearman correlation matrix. Correlation-based clustering was performed by WGCNA. Values followed by different superscript letters are statistically different (*P* < 0.001) according to a random permutation test.

A commonality among all genotypes, however, is the lack of correlation of 16- and 18-carbon FAs with the majority of the cuticular wax metabolites. Notably, these FAs are found either as singleton, nonclustered metabolites (B73, Mo17, Mo17×B73) or within a single small cluster (B73×Mo17) (Fig. 2, D and E; Supplementary Fig. S2), consistent with the fact that the metabolism of these shorter-chain fatty acids is distinct from the other cuticular wax metabolites, i.e. hydrocarbons and VLCFAs. For example, 18-carbon fatty acyl-CoAs serve as the initial precursors for elongation to generate VLCFAs (Bach and Faure 2010), and as building blocks in the assembly of numerous complex lipids, including membrane glycerolipids (Sato and Awai 2017), phospholipids (Nakamura 2017), and sphingolipids (Michaelson et al. 2016).

### Identification of the signature metabolites that are primary contributors to compositional variation in the cuticular wax metabolome

Signature cuticular wax metabolites (or biomarkers) were identified via multivariate Partial Least Squares-Discriminant Analysis (PLS-DA). This statistical strategy is commonly applied to metabolite data for classification analysis and biomarker selection (Worley and Powers 2012; Bartel et al. 2013). We used this strategy herein to identify the signature metabolites that most contribute to the variation in the cuticular wax metabolome as modulated by the change in the silk microenvironment or by the silk genotype. In this study, two supervised PLS-DA regression models were constructed, with the explanatory variables (*X*) being the concentrations of individual cuticular wax metabolites and the response variable (*Y*) being either genotype (Supplementary Fig. S3, A and C) or silk section (Supplementary Fig. S3, B and D). The corresponding weight plots that illustrate the contributions of each metabolite in discriminating among genotypes or silk sections are presented in Supplementary Fig. S3, C and D, respectively. The metabolites that contribute the most to PLS-DA classification were identified via a variable-importance-in-projection score (VIP > 1; Supplementary Table S5), which is a cumulative measure of the contribution of a given explanatory variable (i.e. metabolite) to a PLS-DA model (Pérez-Enciso and Tenenhaus 2003).

**Figure 3.**
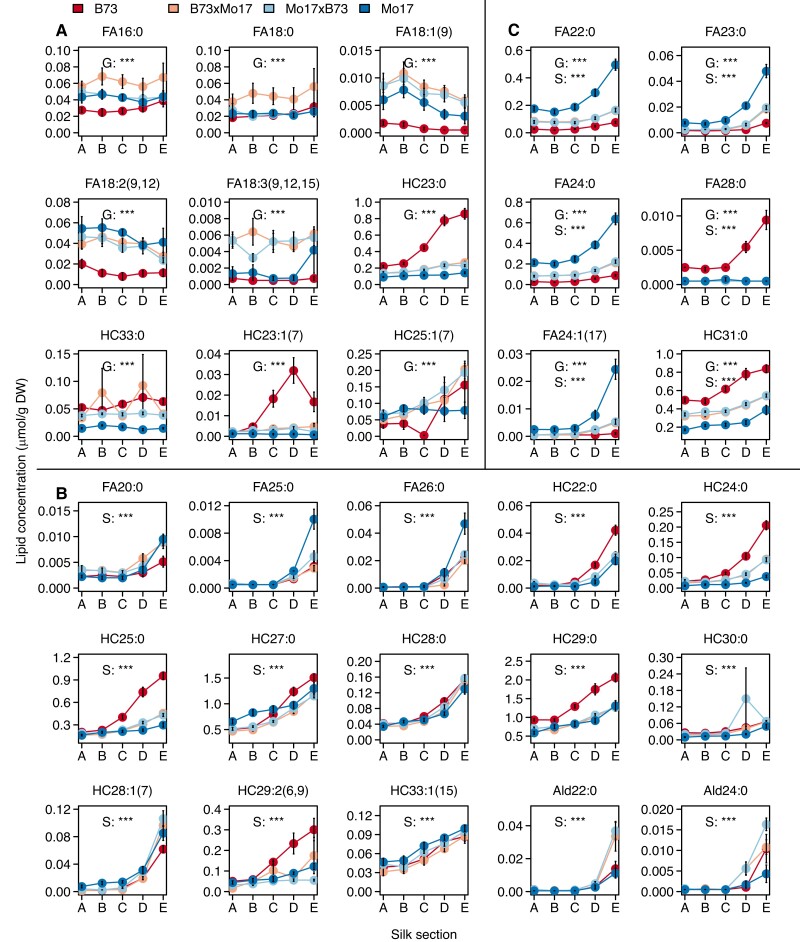
Accumulation patterns of signature cuticular wax metabolites that distinguish among genotypes or among silk sections. Concentrations of signature cuticular waxes that were identified by partial least square discriminant analysis (PLS-DA) as having variable-importance-in-projection (VIP) scores >1. Nine (**A**) and 15 (**B**) of these metabolites contribute to either the observed genotype-based or silk section-based PLS-DA separation, respectively, and an additional six lipid metabolites were selected in both categories (**C**). Two-way ANOVA of the main effects of genotype and silk section were conducted for each metabolite, and statistical significance is noted with asterisks (***, *P* < 0.0001) for genotype (G), silk section (S), or both G and S. The interaction effect, G × S was also evaluated by ANOVA for each metabolite and presented in Supplementary Table S3. Averages ± standard errors from seven to eight biological replicates are reported (*N* = 158) as presented in Fig. 1, with averages connected by colored lines to facilitate visualization. Abbreviations: Ald, aldehyde; DW, dry weight; FA, fatty acid; HC, hydrocarbon.

The PLS-DA classification of cuticular wax metabolome compositions relative to genotype yields two clusters of silk samples for each of the inbred lines, and a third cluster containing the samples from the two hybrids (Supplementary Fig. S3A). The resulting model explained 63% and 66% of the variance in the explanatory (*R*^2^*X*) and response (*R*^2^*Y*) variables, respectively. The predictive accuracy is relatively low for this model (i.e. *Q*^2^*Y* = 63%), due to the inability to correctly classify the metabolite compositions between the two hybrids, B73×Mo17 and Mo17×B73, which express nearly identical cuticular wax metabolomes. However, upon combining the metabolite data from the two hybrids into a single group, the predictive accuracy of the model was improved (*Q*^2^*Y* = 89%) (Supplementary Fig. S3E). As visualized in the PLS-DA scatter plot, the signature wax metabolites (i.e. VIP scores >1) identified in the original PLS-DA model are major contributors to sample classification, primarily differentiating B73 from Mo17, and these two parental lines from the B73×Mo17 and Mo17×B73 hybrids (Supplementary Fig. S3C; black circles). These signature metabolites (10 fatty acids and 5 hydrocarbons) (Fig. 3, A and C) are one third of the total number of detected cuticular wax metabolites but account for two thirds of the observed variance in the response variable (i.e. genotype).

The PLS-DA classification model of metabolome compositions relative to the spatio-temporal silk gradient yields one cluster that is specific to husk-encased sections A and B, and three individual clusters specific to silk sections C, D, and E (*R*^2^*X* = 55%, *R*^2^*Y* = 31%, *Q*^2^*Y* = 28%; Supplementary Fig. S3B). A PLS-DA model that distinguishes silk sections based on husk-encasement status improves the predictive accuracy (*Q*^2^*Y*) from 28% to 79% (Supplementary Fig. S3F), demonstrating the importance of the silk microenvironment in shaping the cuticular wax metabolome. Moreover, the PLS-DA model that classifies the metabolomes by silk sections identified 21 signature metabolites (8 fatty acids, 2 aldehydes, and 11 hydrocarbons) (Supplementary Fig. S3D, black circles), each of which exhibited statistically significant differences in patterns of accumulation among the silk sections (Fig. 3, B and C). In combination, these signature metabolites account for 75% of the variance observed among the silk sections. Notably, six metabolites (4 saturated fatty acids, 1 unsaturated fatty acid, and 1 alkane) were selected as signature metabolites that contribute to the variation observed both across different genotypes and different silk sections (Fig. 3B). Collectively, these quantitative statistical analyses demonstrate the importance of both the silk microenvironment and the silk genotype to determining the cuticular wax composition and further identify that the accumulation of many cuticular waxes, especially the VLCFAs and hydrocarbons, are differentially influenced by these two factors.

The signature metabolites include six pairs of saturated or unsaturated hydrocarbons and fatty acids (i.e. either *HC*_(*2n-1*)*:0*_ and *FA_2n:0_*, or *HC_(2n-1):1_* and *FA_2n:1_* metabolites) that are derived from the same VLCFA-CoA precursor. There is a striking difference in the accumulation of these cuticular VLCFAs as compared to the hydrocarbons in the two inbreds. Namely, compared to B73 silks, the cuticular VLCFAs of 22 to 26 carbons are 2- to 13-fold more abundant in Mo17, whereas the metabolically related hydrocarbons (i.e. of 21 to 25 carbons) are 2- to 13-fold less abundant in Mo17 (Supplementary Fig. S4). These distinct accumulation patterns of cuticular hydrocarbons and VLCFAs reveal a difference in the cuticular wax-producing network between the two inbreds, likely caused by preferential allocation of VLCFA-CoA precursors to either decarbonylation (generating hydrocarbons) or hydrolysis (generating VLCFAs).

### Metabolic partitioning of VLCFA-CoA precursors determined from the concentrations of VLCFA-CoAs, total free VLCFAs, and complex lipid-associated VLCFAs

**Figure 4.**
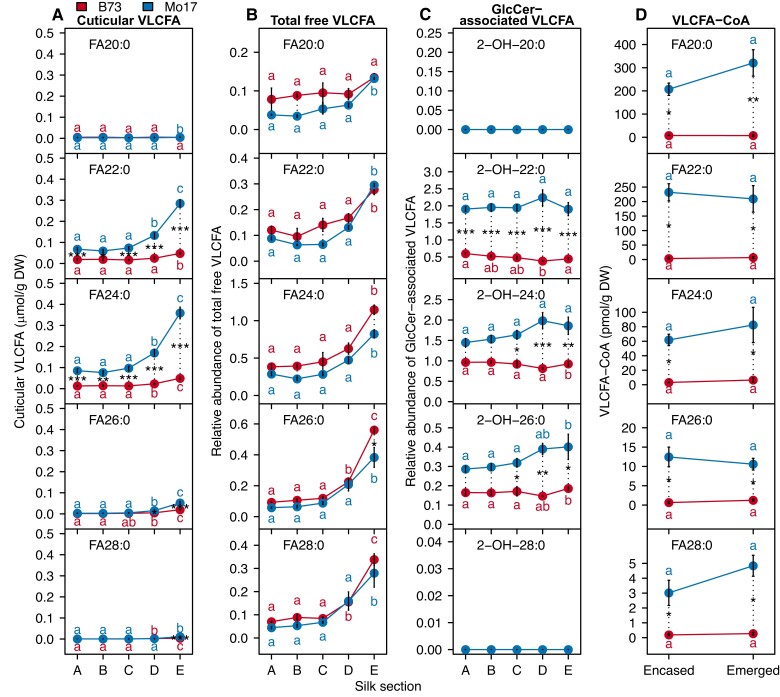
Accumulation of four VLCFA pools along the silk length. Concentrations of individual cuticular VLCFAs (**A**), total free VLCFAs (**B**), glucosylceramide (GlcCer)-associated VLCFAs (**C**), and VLC fatty acyl-CoAs (VLCFA-CoAs) (**D**) in different silk sections of inbred lines B73 (red data points) and Mo17 (blue data points). Cuticular VLCFA concentrations are reported as µmol/g dry weight and VLCFA-CoA concentrations are reported as pmol/g dry weight. Total free VLCFAs and GlcCer-associated VLCFAs are reported as relative abundance in comparison to the phosphatidylcholine internal standard that was added to each sample during lipid extraction. For each genotype, different letters associated with data points indicate statistical differences among silk sections (*P* < 0.05; Tukey's HSD). The genotype difference for each metabolite per silk section is represented by the length of the dotted lines. Asterisks denote statistical differences between genotypes at a specific position along the silk length (***, *P* < 0.0001; **, *P* < 0.001; *, *P* < 0.05). Averages ± standard errors from three to four biological replicates (two ears pooled from two separate plants per replicate) are reported for the VLCFA pools; *N* = 35. Abbreviations: DW, dry weight; FA, fatty acid; HSD, honestly significant difference; VLCFA, very long-chain fatty acid; VLCFA-CoA, very long-chain fatty acyl-CoA.

The VLCFA-CoAs that contribute to the assembly of the silk cuticular waxes are products of the endoplasmic reticulum-associated fatty acid elongase (Yeats and Rose 2013). But these VLCFA-CoAs can also be converted to free VLCFAs or can be utilized to assemble other complex lipids, e.g. phospholipids, neutral lipids, and sphingolipids. Therefore, to address this metabolic partitioning of the VLCFA-CoAs, we extracted and analyzed total silk lipids (Fig. 4, B and C) in parallel with the preparation of cuticular wax extracts from silks of B73 and Mo17 inbreds (Fig. 4A). These lipids were profiled by liquid chromatography-mass spectrometry (LC-MS), which identified the occurrence of free VLCFAs and VLCFAs that were associated with complex lipids. The free VLCFAs quantified in these analyses combined both cellular and cuticular VLCFA pools. These total free VLCFAs were of 20- to 34-carbon chain lengths (Supplementary Table S6). The VLCFA-containing lipids were predominantly associated with the sphingolipids, specifically three glucosylceramide (GlcCer) species that contain FA_22:0_, FA_24:0_, or FA_26:0_ (Supplementary Table S6). Although VLCFAs were also detected as associated with phosphatidylcholine and triacylglycerol, these VLCFAs were in trace amounts, and therefore ignored.

Next, we sought to explore the relationship between different VLCFA pools and cuticular VLCFAs. The total free VLCFAs increased in abundance from the base to the tip of the silks (Fig. 4). This increase correlated with the cuticular VLCFAs on silks of both B73 (Pearson correlation coefficients, 0.65 to 0.92) and Mo17 (0.87 to 0.94). In contrast, no such correlation was observed with the GlcCer-associated VLCFAs for either genotype (Supplementary Table S7).

Because VLCFA-CoAs are the substrates for cuticular wax biosynthesis, we also profiled these fatty acyl-CoA pools in two sections of the silks, the husk-encased (i.e. represented by sections A–C, Fig. 1A) and the emerged silks (i.e. represented by sections D and E, Fig. 1A) of B73 and Mo17 plants. Fatty acyl-CoAs of 16- and 18-chain lengths comprised 80% to 90% of the acyl-CoA pools, and these were more abundant in Mo17 silks, particularly in the emerged portions of the silks (Supplementary Fig. S5 and Supplementary Table S8). These analyses quantify VLCFA-CoAs of up to 34-carbon chain length (Supplementary Fig. S5), including the chain lengths that are precursors of the cuticular wax metabolites (i.e. 20 to 28 carbons) (Fig. 4D). The spatio-temporal accumulation of individual VLCFA-CoAs along the silk length is not statistically correlated with the corresponding cuticular wax products (Supplementary Table S9). On the other hand, VLCFA-CoA accumulation differs between inbreds. In the husk-encased silk sections, the 20- to 28-carbon VLCFA-CoAs accumulated at 16- to 68-fold higher levels in Mo17 than B73, and in the emerged sections of silks these VLCFA-CoAs were between 8- and 44-fold higher in Mo17 (Fig. 4D). This genotypic difference in VLCFA-CoA accumulation is also observed for cuticular VLCFAs.

### Cuticular hydrocarbon:VLCFA ratios vary among genotypes and across acyl chain lengths

Three sets of analyses were conducted to assess the metabolic commitment of VLCFA-CoA precursors to the cuticular wax-producing network. First, we examined the ratios between hydrocarbons (*HC_2n-1:0_*) and the corresponding cuticular VLCFAs (*FA_2n:0_*), both of which are derived from the same precursors (*FA_2n:0_-CoA*). Second, we sought to unravel the product-precursor relationship within the cuticular wax-producing network by studying the associations between *FA_2n:0_*-CoA concentrations and the cuticular wax hydrocarbon:VLCFA ratios (i.e. *HC_2n-1:0_:FA_2n:0_*). Finally, this interrogation was further expanded by integrating the associations of the *HC_2n-1:0_:FA_2n:0_* ratios with the other two VLCFA pools (total free VLCFAs and GlcCer-associated VLCFAs), which are also derived from VLCFA-CoAs.

**Figure 5.**
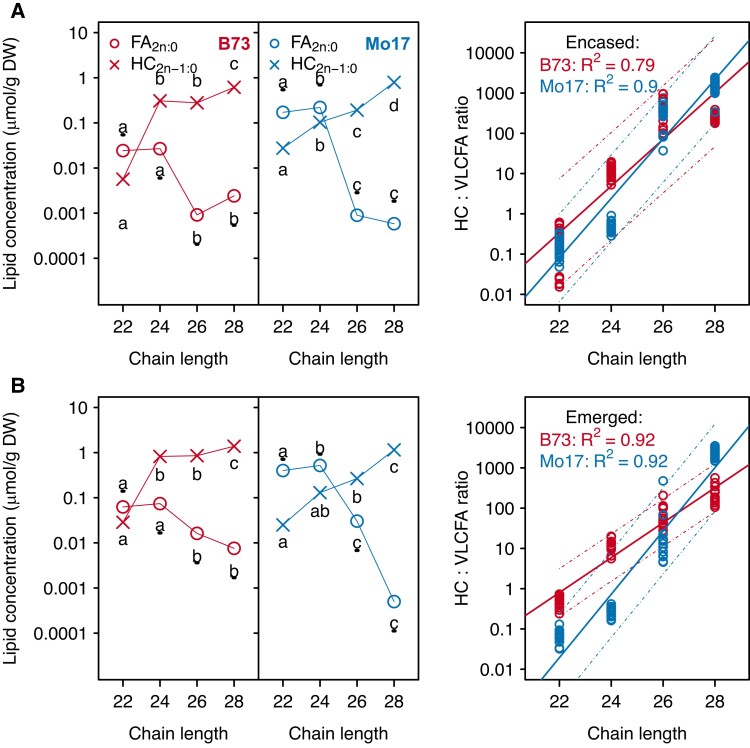
Accumulation of cuticular hydrocarbon products and the metabolically related cuticular VLCFAs, and the regression of the hydrocarbon:VLCFA on chain length for inbreds B73 and Mo17. Concentrations (log-scaled) of cuticular hydrocarbons (*HC_2n-1:0_*) and VLCFAs (*FA_2n:0_*) were analyzed from husk-encased silks (**A**) and from silks that had emerged from the husks (**B**) of the inbreds B73 (red data points) and Mo17 (blue data points). Averages ± standard errors from seven to eight biological replicates as presented in Fig. 1 are reported for the metabolite concentrations. Different letters associated with data points from the same metabolite class indicate a statistically significant difference between acyl chain lengths (*P* < 0.05; Tukey's HSD test); letters associated with VLCFAs are underlined. In the regression, the prediction intervals for the regression models are indicated by the dashed lines for each inbred. Abbreviations: DW, dry weight; FA, fatty acid; HC, hydrocarbon; HSD, honestly significant difference; VLCFA, very long-chain fatty acid.

Among the factors that were considered in this study, the cuticular *HC_2n-1:0_:FA_2n:0_* ratios were most influenced by the carbon chain lengths of these metabolites. In the four genotypes examined, the saturated cuticular VLCFA (*FA_2n:0_*) of each *HC_2n-1:0_:FA_2n:0_* pair decreased in abundance with increasing carbon chain length, concomitant with an increase in the corresponding alkane product (*HC_2n-1:0_*) (Fig. 5 and Supplementary Fig. S6). Indeed, regression analysis of the *HC_2n-1:0_:FA_2n:0_* ratio confirms a linear increase in the value of this ratio as the chain length increases from 22 to 28 carbon atoms (*P*-values <0.0001, *R*^2^ ≥ 0.79; Fig. 5; Supplementary Fig. S5), and this is independent of silk husk-encasement status or genotype. In addition, the abundance of VLCFA-CoA precursors, total free VLCFAs and GlcCer-associated VLCFAs decrease with increasing acyl chain length (Fig. 4 and Supplementary Table S6). Specifically, with increasing acyl chain length, regardless of the genotype or silk micro-environment, VLCFA-CoA precursors (*FA_2n:0_*-CoA) are negatively correlated with the corresponding *HC_2n-1:0_:FA_2n:0_* ratio (correlation coefficients < −0.8), negatively correlated with *HC_2n-1:0_* (correlation coefficients < −0.6), and positively correlated with cuticular *FA_2n:0_* (correlation coefficients > 0.7) (Supplementary Table S9). These findings suggest that VLCFA-CoAs of longer acyl chain lengths are increasingly recruited for hydrocarbon biosynthesis, instead of being converted to free VLCFAs and subsequently deposited in the cuticle.

Because cuticular wax production is indirectly associated with total free VLCFAs and with GlcCer-associated VLCFAs, a more rigorous statistical approach, Bayesian model selection, was employed to evaluate the impact (or lack thereof) of these two VLCFA pools on the cuticular *HC_2n-1:_*_0_:*FA_2n:0_* ratio. Specifically, while keeping metabolite chain length (*2n*) in the baseline model, our goal was to establish the relative importance of the other variables on the *HC_2n-1:_*_0_:*FA_2n:_*_0_ ratio (i.e. total free VLCFAs, GlcCer-associated VLCFAs, genotype, husk-encasement status, and the macroenvironment). The construction of Bayesian regression models and the methods of their comparisons are detailed in Supplementary Methods. Because we did not have a single dataset that integrates the factors of both VLCFA pools and the macroenvironments (i.e. 2014 and 2015 growing years), we performed two Bayesian Model Comparisons, examining the impact of the VLCFAs pools on the *HC_2n-1:_*_0_:*FA_2n:0_* ratio (i.e. VLCFA model selection) or the impact of the difference in the macroenvironment on this ratio (i.e. Macroenvironment model selection).

To assess the impact of total free VLCFAs and GlcCer-associated VLCFAs on the *HC_2n-1:_*_0_:*FA_2n:0_* ratios, we considered three Bayesian regression models: VLCFA Model 1, which includes total free VLCFAs; VLCFA Model 2, which includes GlcCer-associated VLCFAs; and VLCFA Model 3, which includes both total free and GlcCer-associated VLCFAs (Table 2). The predictor variables included in each model were selected by preliminary Bayesian Model Comparison (see Supplementary Methods; Supplementary Tables S10–S12) from the variables: genotype, husk-encasement status, and metabolite chain length, as well as those associated with two-way and three-way interaction terms among these predictor variables. Subsequently, a second round of Bayesian Model Comparison was performed to compare a model without VLCFA variables (Baseline Model) to VLCFA Models 1, 2, and 3. The resultant Bayes factors demonstrate a substantial increase in the ability of each VLCFA model to explain patterns of *HC_2n-1:_*_0_:*FA_2n:0_* ratios when incorporating either or both VLCFA pools (Table 2). These findings demonstrate that the allocation of VLCFA-CoAs to biological processes different from cuticular wax production strongly impact cuticular *HC_2n-1:_*_0_:*FA_2n:0_* ratios.

**Table 2.** Impacts of total free VLCFAs and glucosylceramide (GlcCer)-associated VLCFAs on the cuticular hydrocarbon: VLCFA ratios determined by Bayesian model comparison

Baseline model	VLCFA models^a^	Examined terms	Bayes factor^b^
Genotype (*g*) + Encasement status (*k*) + Acyl chain length (*n*) + (*g* × *k*) + (*k × n*) + (*g × k × n*)	VLCFA model 1: *g* + *k* + *n +* (*g* × *k*) + (*k × n*) + (*g × k × n*) + [**free VLCFAs** (*ffa*) + (*ffa × g*) + (*ffa × k*) + (*ffa × n*) + (*ffa × k × n*)]	Total free VLCFAs (*ffa*) and the interaction effects with *g*, *k*, and *n*	1.88 × 10^7^
	VLCFA model 2: *g* + *k* + *n +* (*g* × *k*) + (*k × n*) + (*g × k × n*) + [**GlcCer-associated VLCFAs** (*gfa*) + (*gfa × g*) + (*gfa × n*)]	GlcCer-associated VLCFAs (*gfa*) and the interaction effects with *g* and *n*	8.83 × 10^6^
	VLCFA model 3: *g* + *k* + *n +* (*g* × *k*) + (*k × n*) + (*g × k × n*) + [*ffa* + *gfa* + (*gfa × n*)]	Total free VLCFAs (*ffa*) and GlcCer-associated VLCFAs (*gfa*), and interaction effects with *g*, *k*, and *n*	8.27 × 10^6^

The allocation of VLCFA-CoA precursors within the cuticular wax-producing network was represented by the linear regression of cuticular *HC_2n-1_:FA_2n_* ratio versus acyl chain length, *2n*. Bayesian model comparison was performed on metabolomics data collected during the 2015 growing season with total free VLCFAs and GlcCer-associated VLCFAs profiled from lipids extracted from the entire silk tissue.

^a^For each VLCFA model, the main effect of the examined term and the associated interaction effects were added to the Baseline model as justified in Supplementary Tables S10–S12.

^b^Bayes factors were calculated as the ratio of the likelihood between a VLCFA model (numerator) and the Baseline model (denominator). A Bayes factor >3.2 indicates that the addition of the examined terms to the Baseline model resulted in significantly better fit to the data.

Finally, using combined data collected from the two growing years, we queried the effect of the macroenvironment on *HC_2n-1:_*_0_:*FA_2n:0_* ratios in the context of the different genotypes and silk encasement status (Table 3, Macroenvironment model selection). Because the VLCFA pools were not measured in the first growing year, total free VLCFAs, GlcCer-associated VLCFAs and the related interaction terms were not included in this modeling effort. The genotype factor exhibited the strongest impact on hydrocarbon:VLCFA ratios in the combined dataset (Bayes factor = 1.01 × 10^110^, Table 3, Macroenvironment model selection). Change in the macroenvironment (Bayes factor = 2.35 × 10^33^) and microenvironment (Bayes factor = 7.76 × 10^28^) also impacted the *HC_2n-1:_*_0_:*FA_2n:0_* ratios, although to a lesser extent. Similarly, these quantitative modeling analyses indicate that the two-way interactions between genotype and microenvironment, and between genotype and macroenvironment also play a role in determining the *HC_2n-1:_*_0_:*FA_2n:0_* ratio dynamics along the silk length (Table 3). In contrast, the interaction between the micro- and macroenvironments do not. Collectively, these analyses quantitatively illustrate the complexity of factors that influence the relationships between precursors, intermediates and products of a complex metabolic process, such as cuticular wax biosynthesis. The quantitative relationships defined by this study involve commitment of VLCFA-CoA precursors among multiple competing metabolic branches of the cuticular wax-producing network that are represented by the steady state levels of cuticular hydrocarbons, cuticular VLCFAs, total free VLCFAs, and GlcCer-associated VLCFAs. Our analyses indicate that with increasing acyl chain length, the hydrocarbon-producing pathway is prioritized over the other competing branches.

**Table 3.** Impact of genotype, husk-encasement status, and growing year on the cuticular *HC_2n-1_:FA_2n_* ratio determined by Bayesian model comparison

Baseline model	Macroenvironment models^a^	Examined term	Bayes factor^b^
Genotype (*g*) + Encasement status (*k*) + Growing year (*y*) + Acyl chain length (*n*) + (*g* × *k*) + (*g* × *y*) + (*g* × *n*) + (*k* × *n*) + (*k* × *y*) + (*y* × *n*) + (*g* × *k* × *n*) + (*g* × *y* × *n*) + (*k* × *y* × *n*) + (*g* × *k* × *y* × *n*)	*k* + *y* + *n* + (*k* × *y*) + (*k* × *n*) + (*y* × *n*) + (*k* × *y* × *n*)	Genotype (*g*)	1.01 × 10^110^
	*g* + *y* + *n* + (*g* × *y*) + (*g* × *n*) + (*y* × *n*) + (*g* × *y* × *n*)	Growing year (*y*)	2.35 × 10^33^
	*g* + *k* + *n* + (*g* × *k*) + (*g* × *n*) + (*k* × *n*) + (*g* × *k* × *n*)	Encasement status (*k*)	7.76 × 10^28^
	*g* + *k* + *y* + *n* + (*g* × *n*) + (*k* × *n*) + (*k* × *y*) + (*y* × *n*) + (*k* × *y* × *n*)	*g* × *k*	8.80 × 10^7^
	*g* + *k* + *y* + *n* + (*g* × *k*) + (*g* × *n*) + (*k* × *n*) + (*k* × *y*) + (*y* × *n*) + (*g* × *k* × *n*) + (*k* × *y* × *n*)	*g* × *y*	3.52 × 10^6^
	*g* + *k* + *y* + *n* + (*g* × *k*) + (*g* × *y*) + (*g* × *n*) + (*k* × *n*) + (*y* × *n*) + (*g* × *k* × *n*) + (*g* × *y* × *n*)	*k* × *y*	5.49 × 10^−9^

The allocation of VLCFA-CoA precursors within the cuticular wax-producing network was represented by the linear regression of cuticular *HC_2n-1_:FA_2n_* ratio versus acyl chain length, *2n*. Bayesian model comparison was performed on the combined metabolomics datasets gathered from tissue grown in 2014 and 2015.

^a^For each modified model, the main effect of the examined term and the associated interaction effects were removed from the Baseline model.

^b^Bayes factors were calculated as the ratio of likelihood between the Baseline model (numerator) and modified models (denominator). A Bayes factor >3.2 indicates that the removal of the examined terms from the Baseline model resulted in significantly worse fit to the data. Examined terms are therefore listed in descending order of importance to the model.

## Discussion

### Maize silks as a unique system to study the dynamics of the cuticular wax-producing network

In this study, we utilized cuticular wax profiling data gathered along the length of maize silks from different genetic backgrounds to deduce and quantitatively compare the dynamics of the metabolic network that supports cuticular hydrocarbon accumulation. We investigated the cuticular wax metabolomes of the maize B73 and Mo17 inbred lines, and the corresponding reciprocal hybrids. Genotypic variation of cuticular wax compositions occur primarily between the two inbred parents (i.e. B73 versus Mo17) and between the inbred parents and the hybrids, but not between the reciprocal hybrids, B73×Mo17 and Mo17×B73. While some plant molecular phenotypes (e.g. chloroplast thylakoid lipid composition and localization; Dueñas et al. 2017) and gene expression profiles (Gonzalo et al. 2007; Swanson-Wagner et al. 2009) can differentiate between these reciprocal hybrids, such distinctions are less pronounced than the differences that occur between the parental inbred lines (Paschold et al. 2012; Baldauf et al. 2016; Marcon et al. 2017). The remainder of the discussion is therefore focused on the cuticular wax metabolomes and the apparent variation in the underlying hydrocarbon-producing pathway between the two inbred parents.

Maize silks provide a unique platform for assessing the dynamics of cuticular wax production, in the absence of complex metabolic flux analysis approaches. With few exceptions, the vast majority of characterizations of the cuticular wax biosynthesis pathway have been conducted from single time-point comparisons of metabolomes profiled from different genetic backgrounds (Bianchi et al. 1979; Koornneef et al. 1989; Hannoufa et al. 1993; Jenks et al. 1996; Post-Beittenmiller 1996; Perera et al. 2010; Xue et al. 2017; Trivedi et al. 2019). In contrast, in this study cuticular wax profiling captures differences in surface metabolite accumulation of presumed precursors, intermediates, and products of the pathway along a spatio-temporal gradient that includes the developmental progression of silks (Fuad-Hassan et al. 2008), and the transition in microenvironment as portions of the silks changed encasement status from husk-encased to husk-emerged in two separate macroenvironments. Similar spatio-temporal studies have identified metabolic shifts in primary, secondary, and lipid metabolism along the developmental gradient that integrates cell division and cell expansion in Arabidopsis (*Arabidopsis thaliana*), wheat (*Triticum aestivum*) and maize (Pick et al. 2011; Allwood et al. 2015; Busta et al. 2017; Zhou et al. 2019; Bourgault et al. 2020).

### The silk microenvironment significantly impacts the cuticular wax metabolome and the hydrocarbon-producing pathway

Emergence of the silks from the encasing husk-leaves has previously been shown to induce increased accumulation of cuticular hydrocarbons in different maize genotypes (Yang et al. 1992; Miller et al. 2003; Perera et al. 2010; Loneman et al. 2017; Dennison et al. 2019). However, in each of these prior studies that compared the cuticular wax metabolome between husk-encased and emerged portions of silks, the change in microenvironment (i.e. silk encasement status) was overlaid with a confounding developmental gradient, particularly that of an acropetal decrease in cellular elongation within the husk-encased silks and cessation of elongation within emerged silks at the time of tissue sampling (Fuad-Hassan et al. 2008). This study shows that the change in microenvironment per se drives the observed change in cuticular wax composition, as well as the ratios between cuticular hydrocarbons and corresponding cuticular VLCFAs. A recent transcriptomic atlas for maize silks has identified this microenvironmental change induces differential expression for many genes involved in cuticular wax biosynthesis, especially for VLCFA metabolic processes, and more broadly for genes involved in responding to biotic and abiotic stresses (McNinch et al. 2020). The increase in cuticular hydrocarbon accumulation that is observed in emerged silks of B73, the B73×Mo17 and Mo17×B73 hybrids and, to a lesser extent, Mo17, may provide protection against drought, similar, for example, to the observed increase in alkane accumulation upon drought stress that occurs in soybean (*Glycine max*) and sesame (*Sesamum indicum*) (Kim et al. 2007a, b). These differences in cuticular wax composition may also be caused by additional environmental cues imposed upon husk-encased versus emerged silks (e.g. light, oxygen concentration, and other biotic and abiotic factors) as occurs in other plant organs (Lewandowska et al. 2020). For example, a number of prior studies have indicated that increased light exposure (von Wettstein-Knowles et al. 1980; Jenks et al. 1994; Rhee et al. 1998) or enhanced UV-B radiation (Tafolla-Arellano et al. 2018) alters cuticular wax composition, especially for alkane constituents (Steinmüiller and Tevini 1985; Tevini and Steinmüller 1987; Gordon et al. 1998). Indeed, recent studies have demonstrated that phytochrome-mediated light signaling regulates cuticle deposition in maize leaves (Qiao et al. 2020a, b).

### Supervised and unsupervised multivariate analyses identified key factors that impact the cuticular wax-producing network

In metabolomic studies, correlation networks are often used to identify metabolites that are co-regulated in response to perturbations caused by disease, chemical and other environmental treatments, or genetic modifications (DiLeo et al. 2011; Fukushima et al. 2011; Papazian et al. 2016; Angelovici et al. 2017; Desmet et al. 2021). Such correlation networks have been constructed from nontargeted metabolomics data gathered from different maize inbred lines, which capitulate pathways of primary metabolism in leaves (Toubiana et al. 2016; Zhou et al. 2019) and kernels (Rao et al. 2014). However, the correlative relationships of metabolites within targeted metabolomes are more complex, as queried metabolites are often from highly related pathways, and therefore the accumulation patterns of most metabolites are inherently correlated. Even so, such correlation networks have successfully described changes in metabolic states due to changes in development-, genotype-, or stress-specific impacts (Steuer 2006; Sawada et al. 2009; Warth et al. 2015; Li et al. 2016; French et al. 2018). In our correlation analyses of metabolites involved in the cuticular wax-producing network, hydrocarbon products (*HC_2n-1_*) and the metabolically related cuticular VLCFAs (*FA_2n_*), separated into different clusters within the correlation network in Mo17, whereas these metabolites are highly correlated in B73. The two major branches of the cuticular wax-producing network, i.e. the hydrocarbon-producing and the cuticular VLCFA-producing sub-pathways, compete for VLCFA-CoA precursors. Our findings demonstrate that interactions between these two branches are determined by the genetic background.

The combination of metabolite profiling and subsequent statistical analyses allows for the identification of signature metabolite biomarkers that represent changes in metabolic status induced by genetic or environmental perturbations or by tissue development (Fernandez et al. 2016; Hong et al. 2016). For example, metabolite biomarkers have successfully uncovered variations within complex pathways among different oat (*Avena sativa*) cultivars (Pretorius et al. 2021), or in response to developmental or environmental stimuli in Arabidopsis (Sulpice et al. 2009), rice (*Oryza sativa*) (Tarpley et al. 2005), maize (Obata et al. 2015; Luo et al. 2019), *Brassica nigra* (Papazian et al. 2016), and wheat (Kang et al. 2019). Herein, we identified 30 cuticular wax-derived signature metabolites that include six pairs of hydrocarbons and the metabolically related cuticular VLCFAs. The accumulation patterns of these signature metabolites along the silk length demonstrates that the cuticular wax-producing network is enhanced along the spatio-temporal developmental gradient of the silks, especially at the transition of the microenvironment as silks emerge from husk-encasement. Furthermore, similar to the findings from correlation network analysis, a comparison of the concentrations of the signature hydrocarbon–VLCFA pairs between B73 and Mo17 demonstrate substantial genetic difference in the relationship between the VLCFA-CoA precursor and the corresponding cuticular wax product, i.e. cuticular hydrocarbon or cuticular VLCFA. Thus, these combined supervised and unsupervised multivariate analyses of metabolomics data not only provide critical insights into the factors that impact cuticular wax accumulation and composition but also allow us to probe the relationships among the individual metabolite conversions within the cuticular wax-producing network.

### Factors impacting the product–precursor relationship of the cuticular wax-producing network

Unlike the hydrocarbon end-products of the cuticular wax biosynthesis pathway that only accumulate extracellularly on the silk surface (Perera et al. 2010; Loneman et al. 2017), VLCFAs accumulate both on the silk surface as the free VLCFA components of the cuticular waxes, and intracellularly as precursor VLCFA-CoAs, free VLCFAs (possibly carried by lipid transfer proteins) (Lerche and Poulsen 1998; Carvalho and Gomes 2007; Deeken et al. 2016) or associated with complex lipids, e.g. discrete glycerolipids (Michaelson et al. 2016) and sphingolipids (Lynch and Dunn 2004). In this study, the complex-lipid associated VLCFAs were recovered as components of three specific cellular GlcCer species harboring a single hydroxylated C22-, C24-, or C26-VLCFA moiety, and as trace amounts of triacylglycerol. Here we show that the concentrations of individual VLCFAs that accumulate on the silk surface are highly correlated with the concentrations of corresponding total free VLCFAs.

Although VLCFA-CoAs are the precursors for fatty acid-derived cuticular wax metabolites (Yeats and Rose 2013), the relationship between cellular VLCFA-CoA pools and cuticular wax abundance has scarcely been studied, possibly due to the technical challenges associated with identifying and quantifying VLCFA-CoAs even at acyl chain lengths of up to 22 carbon atoms (Reina-Pinto et al. 2009). In the current study, VLCFA-CoAs up to an acyl chain length of 34 carbon atoms were quantified, allowing us to dissect the relationship between VLCFA-CoA precursors and the corresponding cuticular wax products and consider the dynamics of product–precursor relationships.

Product–precursor relationships within the cuticular wax-producing network have previously been inferred from cuticular wax profiles of several systems. These include the relationship between VLCFAs (as presumed precursors) versus alkanes (Busta et al. 2017), alcohols versus wax esters (Lai et al. 2007; Wen and Jetter 2009), and among alkanes, secondary alcohols, and ketones (Wen and Jetter 2009) in Arabidopsis. In the grasses, product–precursor relationships have been determined between primary and secondary diols and esters in the wheat cuticle (Racovita and Jetter 2016), and between unsaturated aldehydes and alkenes of the maize cuticle (Perera et al. 2010). In each case, the accumulation of the precursor metabolites within the cuticle was incorporated into the analysis as proxies of the intracellular precursors, and thereby demonstrating product–precursor relationships amongst these metabolites. We evaluated the product–precursor relationship by querying the association between cuticular wax products and the corresponding cellular VLCFA-CoA precursors, as well as between cuticular waxes and the other VLCFA-containing product pools that are also derived from VLCFA-CoA. Because the cuticular wax-producing network in maize silks predominantly consists of two branches that separately produce hydrocarbons and cuticular VLCFAs, the product–precursor relationships within this network are greatly simplified as compared to other plants or plant organs that also accumulate cuticular alcohols, ketones, and wax esters (Lai et al. 2007; Wen and Jetter 2009; Racovita and Jetter 2016; Busta et al. 2017).

Here, we collected different VLCFA pools as well as cuticular wax metabolomes to characterize the possible metabolic fates of VLCFA-CoA in maize silks, including (i) fatty acid elongation that generates longer chain VLCFA-CoA precursors; (ii) hydrolysis by acyl-CoA thioesterases to generate a cellular pool of free VLCFAs; (iii) sequential hydrolysis and transport for deposition as cuticular VLCFAs; (iv) sequential reduction–decarbonylation reactions that generate hydrocarbons (Lewandowska et al. 2020); (v) assembled into complex lipids to become complex lipid-associated VLCFAs; and (vi) β-oxidation that produces shorter-chain VLCFA-CoAs and acetyl-CoA. Although the VLCFA-CoA levels show minor differences between the husk-encased and emerged silk sections, both cuticular VLCFAs and total free VLCFAs increase in accumulation along the silk length. Thus, VLCFA biosynthesis is likely promoted from the base to the tip of the silks, resulting in increased cuticular wax load (both as VLCFAs and hydrocarbons). Similar associations between fatty acid elongation processes and cuticular wax accumulation along the developmental gradient of the leaves have been observed in Arabidopsis (Busta et al. 2017) and leek (*Allium porrum*) (Rhee et al. 1998).

We further dissected the allocation of VLCFA-CoA precursors between different branches of the cuticular wax-producing network by comparing the abundances of cuticular alkanes (*HC_2n-1:0_*) and cuticular VLCFAs (*FA_2n:0_*). The Bayesian approach that was employed for this dissection has been previously used to predict protein trafficking routes (Paultre et al. 2016), to elucidate genomic and environmental controls over plant phenotypes (Allard et al. 2016; Di Guardo et al. 2017; Montesinos-López et al. 2017; Wang et al. 2019), and to assess gene-to-metabolome associations (Marttinen et al. 2014).

In this study, the Bayesian model testing identified factors that significantly impact the VLCFA-CoA allocation, including the genetic background, the husk encasement status of the silks, the acyl chain length of VLCFA-CoAs, and the allocation of VLCFA-CoAs to metabolic processes that are distinct from cuticle deposition. For example, the ratios between cuticular hydrocarbons (*HC_2n-1_*) and cuticular VLCFAs (*FA_2n_*) are lower in Mo17 than B73, whereas the corresponding VLCFA-CoA precursor pools as well as GlcCer-associated VLCFAs are substantially larger in Mo17 than B73. These findings collectively suggest that the hydrocarbon-producing pathway is less active in Mo17 than B73, possibly due to the competition for VLCFA-CoAs by other pathways that are more active in Mo17 (e.g. GlcCer biosynthesis). Statistical assessment of the *HC_2n-1:0_*:*FA_2n:0_* ratios further demonstrate that environmental cues differentially impact the cuticular wax-producing network between Mo17 and B73. These different modulators that impact VLCFA-CoA allocation between cuticular *HC_2n-1_* and cuticular *FA_2n_* can be further explored either by transgenic strategies to evaluate individual genes that may impact product-precursor relationships, or by studying different maize inbred lines to evaluate the impact of different combinations of alleles on product–precursor relationships.

A key observation made in this study is that regardless of the genetic background or silk micro-environment, the cuticular *HC_2n-1:0_*:*FA_2n:0_* ratio increases and the three VLCFA pools (VLCFA-CoA precursors, total free VLCFAs, and GlcCer-associated VLCFAs) decrease in abundance with increasing acyl chain length. These findings indicate that VLCFA-CoAs, which are common precursors for many biological processes, are preferentially utilized to produce longer-chain hydrocarbons via parallel reduction–decarbonylation reactions. There are several potential mechanisms that could generate this acyl chain length-dependent change in the *HC_2n-1:0_*:*FA_2n:0_* ratio. Specifically in the hydrocarbon-producing pathway, these mechanisms include variations in the substrate preference of (i) the VLCFA-CoA elongation system; (ii) the reductase that converts VLCFA-CoAs to aldehydes; and/or (iii) the decarbonylase that converts aldehydes to hydrocarbons. Indeed, chain length preference has been identified for several of the 26 maize 3-ketoacyl-CoA synthetases (KCSs) responsible for the condensation reaction of the fatty acid elongase system that generates VLCFAs (Campbell et al. 2019; Stenback et al. 2022). Different substrate preferences have been described among Arabidopsis KCS isozymes (Millar and Kunst 1997; Fiebig et al. 2000; Blacklock and Jaworski 2006; Joubès et al. 2008; Kim et al. 2013; Hegebarth et al. 2016, 2017; Huang et al. 2023; Luzarowska et al. 2023). Downstream of the fatty acid elongase system, within the decarbonylative hydrocarbon-forming pathway, Arabidopsis ECERIFERUM1 (CER1) and CER1-LIKE1 proteins each form unique reductase-decarbonylase complexes with CER3 and cytochrome b5, which sequentially convert VLCFA-CoAs to aldehydes and alkanes of different chain lengths (Pascal et al. 2019). These potential mechanisms for altering *HC_2n-1:0_*:*FA_2n:0_* ratios can be tested via heterologous expression of candidate genes within recently engineered yeast systems expressing the maize or Arabidopsis fatty acid elongase complexes (Haslam et al. 2012, 2015; Campbell et al. 2019; Stenback et al. 2022; Batsale et al. 2023).

## Conclusions

In this study, we demonstrate that the combination of spatio-temporal profiling and multivariate analyses is an effective approach for assessing and comparing metabolic networks that are differentially impacted by developmental and environmental perturbations among different genotypes. The study depicts the product–precursor relationship for the cuticular wax-producing network on maize silks through a comprehensive analysis of cuticular wax metabolites, VLCFA-CoA precursor pools, total free VLCFAs, and GlcCer-associated VLCFAs. The compositional dynamics of the cuticular wax metabolome were most impacted by a change in the silk microenvironment and genotype, with minor impact due to the developmental trajectory of the tissue at the time of sampling. These product–precursor relationships are complex and dependent upon the precursor acyl chain length, such that longer chain VLCFA-CoAs are preferentially allocated to hydrocarbon biosynthesis. These findings provide a foundation for dissecting the underlying mechanisms of cuticular hydrocarbon-producing networks in response to changes in microenvironment, ultimately leading to a genotype–metabolite–phenotype understanding of a molecular structure that provides a protective capability to plant surfaces (Yandeau-Nelson et al. 2015).

## Materials and methods

### Nomenclature

Cuticular wax metabolites are represented using a *n:y*(*z*) notation, where *n* represents the number of carbon atoms, *y* the number of double bonds, and *z* the positions of the double bonds in the alkyl chain (Howard and Lord 2003). This study reports three classes of cuticular waxes; very long-chain fatty acids (VLCFAs) that are saturated (*FA_n:0_*) or unsaturated (*FA_n:y_*_(*z*)_), saturated aldehydes (*Ald_n:0_*), and hydrocarbons (HCs), which include saturated alkanes (*HC_n:0_*), and two classes of alkenes: monoenes (*HC_n:1_*_(*z*)_), and dienes (*HC_n:2_*_(*z1, z2*)_). The saturated cellular VLCFA-CoAs are annotated as FA_n:0_-CoA.

### Plant growth, sample collection, and processing

Maize (*Z. mays*) inbred lines B73 and Mo17 and their reciprocal hybrids, B73×Mo17 and Mo17×B73 were grown to maturity at the Iowa State University Agronomy Research Farm (Boone, IA) during the 2014 and 2015 growing years using standard cultivation practices and no supplemental irrigation. Developing ear shoots were covered prior to silking to prevent pollination. Ears were harvested three days after silks had emerged from encasing husk leaves, at a point at which half of the plants in a row were silking (i.e. the mid-silk cohort). Ears were harvested between 11 AM and 1 PM each day and were transported to the laboratory at ambient temperature using insulated chests. The number of biological replicates sampled per genotype was 8 and 12 in the 2014 and 2015 growing years, respectively. Each biological replicate consisted of silks from two ears of similar size and appearance collected from two separate plants and harvested on the same day. Silk samples were prepared as described in McNinch et al. (2020). For each biosample, one sub-sample was flash-frozen in liquid nitrogen and reserved for subsequent total VLCFA profiling and the second sub-sample was immediately subjected to cuticular wax extraction. A random selection of four biological replicates collected in the 2015 growing year was subjected to total cellular lipid analysis.

### Cuticular wax and cellular lipid extraction and derivatization

Cuticular waxes were extracted from fresh silks by immersion for 4 min in 9:1 hexanes:diethyl ether supplemented with the internal standards eicosane (1 µg/ml), nonadecanoic acid (1 µg/ml) and heptadecanol (1 µg/ml), as previously described (Loneman et al. 2017). Extracts were concentrated under a stream of N_2_ gas in a N-EVAP nitrogen evaporator (Organomation Associates, Inc., MA). Extracts were chemically derivatized via transmethylation followed by silylation, as previously described (Loneman et al. 2017). For total lipid analyses, intra- and extracellular lipids were extracted as previously described (Okazaki et al. 2015) from 5 to 8 mg of lyophilized silk tissue that had been pulverized to a fine powder using a Mixer Mill 301 (Retsch GmbH, Germany).

VLCFA-CoAs were extracted from B73 and Mo17 silks, each inbred represented by three biological replicates, using methods adapted from Sun et al. (2006), and all extraction solvents were LC-MS grade (Fisher Scientific, Waltham, MA). Per sample, approximately 300 mg of flash-frozen silk tissue was pulverized with a prechilled mortar and pestle, and transferred and weighed into 2 ml microcentrifuge tubes kept on dry ice until extraction. Weighed samples were spiked with 100 ng of the internal standard, 25:0 Coenzyme A (10 ng/μl pentacosanoyl Coenzyme A ammonium salt, in 1:1:2 1 mM acetic acid/isopropanol/acetonitrile) (Avanti Polar Lipids, Birmingham, AL). The extractions were initiated with 425 µl of freshly made 100 mM potassium phosphate monobasic buffer solution (pH 4.9) followed by the addition of 425 µl isopropanol. Samples were then vortexed for 1 min and placed into an ice-cold sonication water bath for 5 min. The samples were vortexed again for 1 min before the addition of 850 µl acetonitrile and a further 5 min of vortexing to finish the extraction. The extracted samples were centrifuged for 7 min at 16,000 × *g* and supernatants were transferred to new microcentrifuge tubes and dried in a speed-vac concentrator. The dried extracts were then resuspended in 200 μl of 4:1 isopropanol/1 mM acetic acid before being centrifuged 7 min at 16,000 × *g* to remove insoluble sample components and potassium phosphate. The resulting supernatants were filtered with 0.2 micron centrifugal filters (Cat. No. UFC30LG25, Millipore Sigma, Burlington, MA) and subjected to LC-MS/MS analysis.

### Chromatography-mass spectrometric analyses

Gas chromatography of cuticular wax extracts was performed with an HP-5MS cross-linked (5%) diphenyl (95%) dimethyl polysiloxane column (30 m in length; 0.25 mm inner diameter) using helium as the carrier gas, and an Agilent Technologies series 6890 gas chromatograph, equipped with a model 5973 mass detector (Agilent Technologies, Santa Clara, CA). Extracts were introduced to the gas chromatograph via splitless injection of a 2 μl sample and the oven temperature program was as listed for samples that were both transmethylated and silylated (Loneman et al. 2017). Quantification analysis was performed using the AMDIS software package (Stein 1999) with assistance from the NIST Mass Spectral library (http://webbook.nist.gov/chemistry/) for compound identification. Double bond positions within unsaturated hydrocarbon constituents (alkenes) were previously identified via analysis of dimethyl-disulfide (DMDS) adducts, as described and reported in Dennison et al. (2019) and were identified in this study based on peak elution patterns.

Total lipid extracts were analyzed via liquid chromatography quadrupole time-of-flight mass spectrometry on a Waters Xevo G2 Q-TOF MS combined with a Waters ACQUITY UPLC system in both positive and negative ion modes, as previously described (Kimbara et al. 2013). LC-MS data were recorded using MassLynx4.1 software (Waters) and processed using MarkerLynx XS software (Waters). The data matrices were queried against an in-house lipid library (RIKEN, Japan). For data normalization, the original peak intensity values were divided by the internal standard peak intensity value at *m*/*z* 566.382 [M+H]^+^ and *m*/*z* 550.351 [M-CH_3_]^−^ for the positive and negative ion modes, respectively.

Liquid chromatography separation of fatty acyl-CoA constituents was performed with an Agilent Technologies 1290 Infinity II UHPLC instrument equipped with a ZORBAX RRHT Extend-C18 analytical column (80Å pore size; 50 mm in length; 2.1 mm inner diameter; 1.8 µm particle size) that was coupled to a 6470 triple quadrupole mass spectrometer with an electrospray ionization (ESI) source (Agilent Technologies, Santa Clara, CA). A volume of 20 µl of each sample was injected into the LC system. The chromatography was carried out at 40 °C with a flow rate of 0.300 mL/min. All LC-MS/MS solvents used were LC-MS grade (Fisher Scientific, Waltham, MA). Running solvents were A: 15% acetonitrile in water with 0.05% triethylamine and B: 10% water in acetonitrile with 0.05% triethylamine. Initial solvent conditions were 0% B, which was held for 5 min and then increased on a linear gradient to 100% B over 9 min, 100% B was held for 11 min before returning to 0% B over a 5-min linear gradient. A 4-min postrun at 0% B was conducted after each LC-MS/MS acquisition.

Fatty acyl-CoA metabolites were detected using electrospray ionization in positive ionization mode. Nitrogen was used as the service gas for the ion source with a drying gas flow rate of 9 l/min at a temperature of 340 °C, a nebulizing pressure of 30 psi, and a sheath gas flow of 10 l/min at 325 °C. The capillary and nozzle voltages were 4,000 and 1,000 V, respectively. The mass spectrometer was operated in dynamic MRM mode to monitor the neutral loss of *m*/*z* 507 for all target fatty acyl-CoA molecules, as described previously (Sun et al. 2006; Haynes et al. 2008). Mass transitions for the eight detected long-chain and very long-chain fatty acyl-CoA metabolites and the internal standard are provided in Supplementary Table S13. Fatty acyl-CoA LC-MS/MS peak quantification was accomplished using Agilent MassHunter Quantitative Analysis (version 10.0) software (Agilent Technologies, Santa Clara, CA) with all fatty acyl-CoA amounts reported relative to the internal standard (25:0-CoA).

### Quantitative methods

Cuticular wax metabolite concentrations were initially calculated relative to the compatible internal standard (i.e. hydrocarbons quantified relative to eicosane, VLCFAs relative to nonadecanoic acid, and alcohols and aldehydes relative to heptadecanol). Because quantification of each metabolite class did not differ depending on choice of standard, all subsequent quantifications were relative to the eicosane standard. Quantification was performed relative to silk dry weight, to avoid potential differences in metabolite concentrations due solely to differences in water content between genotypes or among silk sections. Representative measurements of silk dry weight were calculated for each silk section (A–E) from surrogate ears harvested on the same days as those used for cuticular wax profiling, and a ratio of dry weight to fresh weight was calculated as a conversion factor to estimate the dry weight of extracted silk samples, as described by Loneman et al. (2017). The measured water content of silk samples was approximately 90% regardless of the position along the silk length, the genetic background, or the growing year.

Detection limits were determined as previously described (Dennison et al. 2019). The detection limits for the datasets collected in growing years 2014 and 2015 were 0.005 and 0.006 µmol g^−1^ dry weight, respectively. Metabolite abundances were censored (Newman et al. 1989) such that concentrations below the detection limit (DL) were assigned a value of DL/2, and concentrations for metabolites that were not detected in a specific condition were assigned the value, DL/10. Consistently low abundance metabolites that were assigned DL/2 or DL/10 values for more than 90% of the samples were removed from the dataset.

### Statistical methods

The raw metabolite data were log-transformed and pareto-scaled. ANOVA was performed to evaluate the effects of genotype, position along the silk, growing year, and cellular free VCLFA and cellular lipid-associated VLCFA content on cuticular wax composition. For each genotype, the accumulation dynamics along the silk length for each lipid class were modeled using linear and quadratic regression. The regression model that best fitted the concentration data for each lipid class was determined by comparing the adjusted *R*^2^ between the two regression models. Tukey's Honestly Significant Difference (HSD) tests were applied for post hoc pairwise comparisons between silk sections either within or among genotypes. These analyses were conducted using the R/stats base package (R Core Team 2022).

PCA was performed using the prcomp() function in the R/stats package and 95% confidence ellipses were constructed using the R/car dataEllipse() function (Fox and Weisberg 2019). Partial least squares-discriminant analysis (PLS-DA) was performed using the R/ropls opls() function that also determined the optimal number of components for the PLS-DA model using 7-fold cross-validation (Thévenot et al. 2015). Variable Importance in Projection (VIP) scores were calculated by the R/ropls getVipVn() function as a cumulative measure of the contribution of each metabolite in distinguishing among genotypes or silk sections (Pérez-Enciso and Tenenhaus 2003). Individual metabolites with VIP scores >1.0 (i.e. with above-average contribution in sample classification) were deemed as metabolite biomarkers that discriminate between classes. The explained fraction of data variance by a PLS-DA model for explanatory and response variables were reported as *R*^2^*X* and *R*^2^*Y*, respectively, and the predictive accuracy of the model was reported as *Q*^2^*Y*. The response variable for PLS-DA is categorical and therefore transformed into a binarized numerical variable for model construction, and *R*^2^*Y* represents the explained variation within the transformed data.

Nonparametric Spearman correlations between every pair of metabolites across silk sections A to E were calculated in each genotype and were compared among genotypes according to Choi and Kendziorski (2009) with modifications that are described in Supplementary Methods. The metabolite correlation networks were constructed based on topological overlap matrices (TOMs) derived from the correlation matrices using the R/WGCNA package (Langfelder and Horvath 2008). Briefly, for each genotype, metabolite clusters were initially determined according to TOMs by hierarchical clustering and then pruned by four independent R functions: pam(), cutreeStatic(), and two cutreeDynamic()s with the “methods” argument specified as either “tree” or “hybrid” (Langelder and Horvath 2008).

The ratios of *HC_2n-1:0_* :*FA_2n:0_* relative to the carbon chain length *2n* were fitted into a linear regression model as the response variable using the R/stat function lm(), with precursor chain length, genotype, silk encasement status, growing year, and the corresponding interaction terms as the explanatory variables. The prediction interval for regression was calculated by R/stat function predict(). Bayesian Model Comparison, using the R/BayesFactor package (Morey and Rouder 2023), was applied to assess the association of the *HC_2n-1:0_* :*FA_2n:0_* ratio with these explanatory variables and two additional explanatory variables (concentration of total free VLCFAs and concentration of GlcCer-associated VLCFAs). Bayesian model construction and comparison are described in detail in Supplementary Methods.

## Supplementary Material

kiae150_Supplementary_Data

## Data Availability

The data underlying this article are available in the article and in its online supplementary material.

## Supplementary data

The following materials are available in the online version of this article.

**Supplementary Methods.** Supplementary statistical methods.

**Supplementary Figure S1**. Accumulation of cuticular monoenes along the spatio-temporal gradient of silks.

**Supplementary Figure S2**. Correlation-based clustering of silk cuticular wax abundance data for hybrids, B73×Mo17 and Mo17×B73.

**Supplementary Figure S3**. Clustering of silk samples by partial least squares discriminant analysis (PLS-DA) based on different genotypes and different silk sections.

**Supplementary Figure S4**. The spatio-temporal accumulation patterns of the metabolically related hydrocarbon (*HC_2n-1:0_*) and VLCFA (*FA_2n:0_*) pairs in inbreds B73 and Mo17.

**Supplementary Figure S5**. Accumulation of fatty acyl-CoAs in the encased and the emerged sections of maize silks from inbreds B73 and Mo17.

**Supplementary Figure S6**. Accumulation of cuticular hydrocarbon products and the metabolically related cuticular VLCFAs, and the regression of the hydrocarbon:VLCFA ratios on chain length for hybrids, B73×Mo17 and Mo17×B73.

**Supplementary Table S1**. Spatio-temporal profiles of cuticular waxes from maize silks of B73, Mo17, and the reciprocal hybrids that were collected in the 2014 and 2015 growing years.

**Supplementary Table S2**. Comparison of models that depict the dynamics of cuticular metabolite accumulation levels among the silk sections in the indicated maize lines.

**Supplementary Table S3**. Two-way and two-way nested ANOVAs that test the main effect of genotype (G), silk section (S), and G × S interaction on each cuticular wax metabolite.

**Supplementary Table S4**. Two-way and three-way ANOVAs of total cuticular waxes and individual lipid metabolite classes.

**Supplementary Table S5**. Variable importance in projection (VIP) score for each lipid in two PLS-DA models built to discriminate genotypes or silk sections based on the cuticular wax metabolome compositions.

**Supplementary Table S6**. Spatio-temporal profiles of cellular very long-chain fatty acids (VLCFAs) from maize silks of B73 and Mo17 collected in the 2015 growing year.

**Supplementary Table S7**. Pearson correlations between total free very long-chain fatty acids (VLCFAs) or glucosylceramide (GlcCer)-associated VLCFAs versus cuticular VLCFAs at individual acyl chain lengths.

**Supplementary Table S8**. Profiles of very long-chain fatty acyl-CoAs from maize silks of B73 and Mo17 collected in the 2015 growing year.

**Supplementary Table S9.** Spearman correlations between cellular very long-chain fatty acyl-CoA precursors (*FA_2n:0_*-CoA), cuticular hydrocarbon (HC_2*n*-1:0_), cuticular very long-chain fatty acids (FA_2*n*:0_), and the ratios between cuticular HC_2*n*-1:0_ and the metabolically related cuticular FA_2*n*:0_.

**Supplementary Table S10.** Identification of the optimal very long-chain fatty acid (VLCFA) Bayesian regression model that incorporated total free VLCFAs as predictors in the model.

**Supplementary Table S11.** Identification of the optimal very long-chain fatty acid (VLCFA) Bayesian regression model that incorporated glucosylceramide (GlcCer)-associated VLCFAs as predictors in the model.

**Supplementary Table S12.** Identification of the optimal very long-chain fatty acid (VLCFA) Bayesian regression model that incorporated total free VLCFAs and glucosylceramide (GlcCer)-associated VLCFAs as predictors in the model.

**Supplementary Table S13.** Mass transitions for the eight long-chain and very long-chain fatty acyl-CoA (VLCFA-CoA) metabolites in maize silks, and that of the internal standard (ISTD), as detected by LC-MS/MS.

## Dive Curated Terms

The following phenotypic, genotypic, and functional terms are of significance to the work described in this paper:

CER3 Gramene: At5g57800
CER3 Araport: At5g57800

fatty acyl-coa CHEBI: CHEBI:37554

hydrocarbon CHEBI: CHEBI:24632

hydrocarbons CHEBI: CHEBI:24632

metabolite CHEBI: CHEBI:25212

VLCFA Gramene: Chebi: 27283
VLCFA Araport: Chebi: 27283

very long-chain fatty acid Gramene: 27283
very long-chain fatty acid Araport: 27283

aldehyde Gramene: Chebi: 17478
aldehyde Araport: Chebi: 17478
