# Supplementary material for: Dynamic relationships among pathways producing hydrocarbons and fatty acids of maize silk cuticular waxes
Source: Plant Physiol. 2024 Mar 27;195(3):2234–55. doi: 10.1093/plphys/kiae150 (PMC11213258; doi:10.1093/plphys/kiae150)
Supplement: kiae150_Supplementary_Data [file kiae150_supplementary_data.zip › PP2023RA01662DR1_Supplemental_Data.pdf]

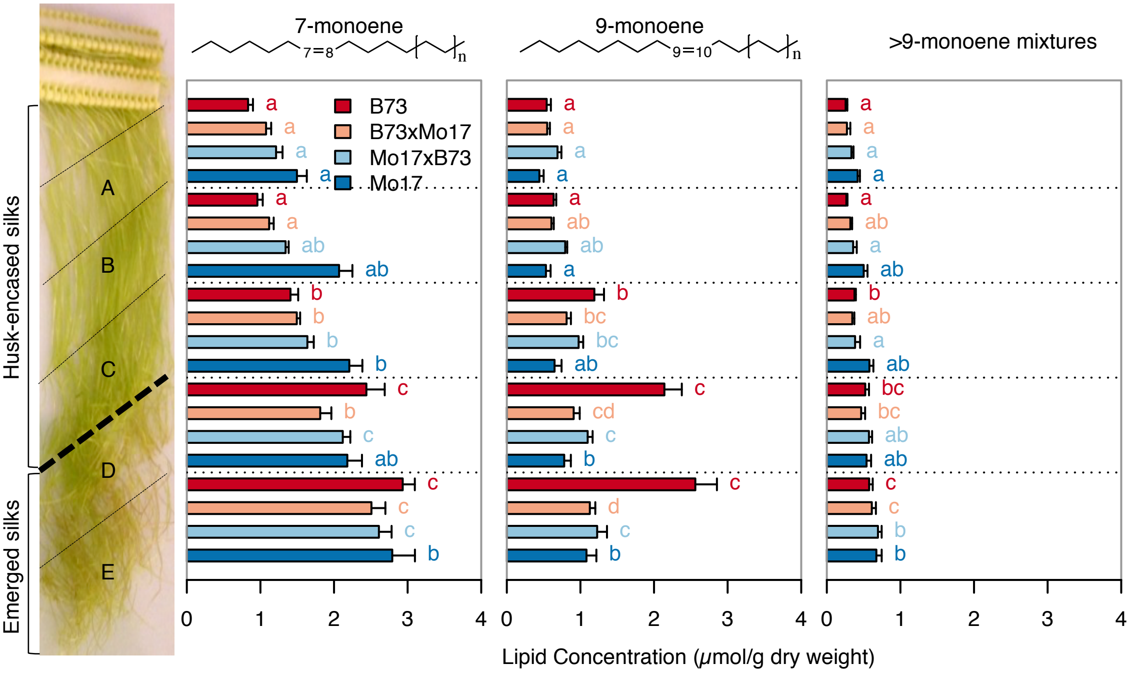

Supplemental Figure S1. Accumulation of cuticular monoenes along the spatio-temporal gradient of silks. Concentrations of cuticular 7-monoenes, 9-monoenes, and other alkenes (a mixture of 14- and 15-monoenes) from different silk sections of inbreds B73 and Mo17, and the reciprocal hybrids. For each genotype, different letters associated with data bars of the same color denote a statistically significant difference in concentrations between silk sections ($p<0.05$; Tukey's Honestly Significant Difference test). Seven or eight replicates were evaluated per combination of genotype and silk section; N=158. Averages ± standard errors are reported.

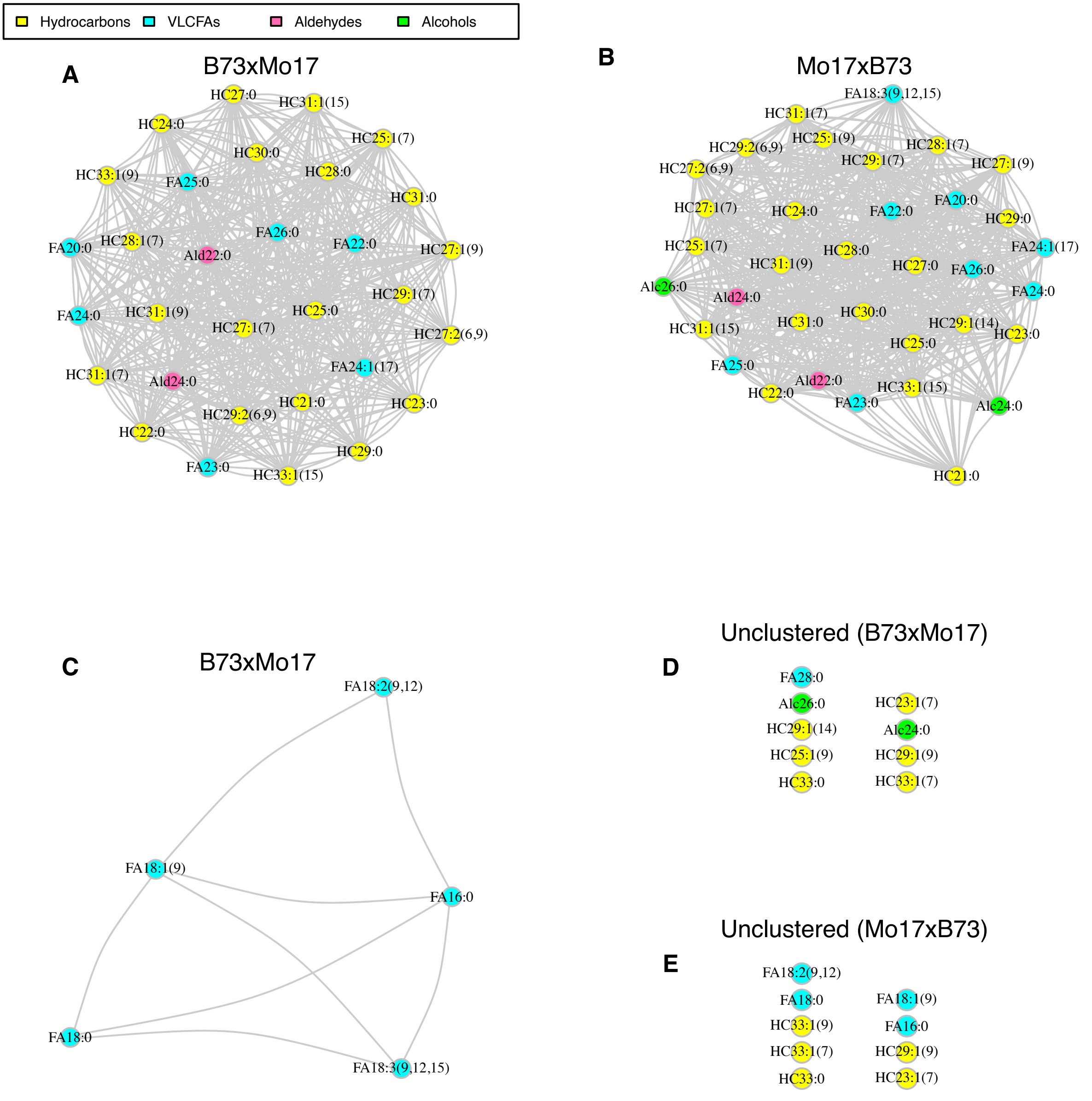

Supplemental Figure S2. Correlation-based clustering of silk cuticular wax abundance data for hybrids, B73×Mo17 and Mo17×B73. Rank-based Spearman correlations were calculated between all pairs of metabolites and used to construct the weighted correlation networks via weight correlation network analysis for B73×Mo17 (**A and C**) and Mo17×B73 (**B**). Pairs of cuticular wax metabolites connected by edges are significantly correlated with coefficients ≥0.5 and reside within the same cluster. Edge length represents correlation strength, with shorter edges representing stronger correlations between metabolites. Unclustered singleton metabolites (**D** and **E**) were not statistically correlated with any other metabolites, or shared correlation values <0.5. The notations for cuticular wax metabolites are described in Methods. Abbreviations: Alc, alcohol; Ald, aldehyde; FA, fatty acid; HC, hydrocarbon; VLCFA, very long-chain fatty acid.

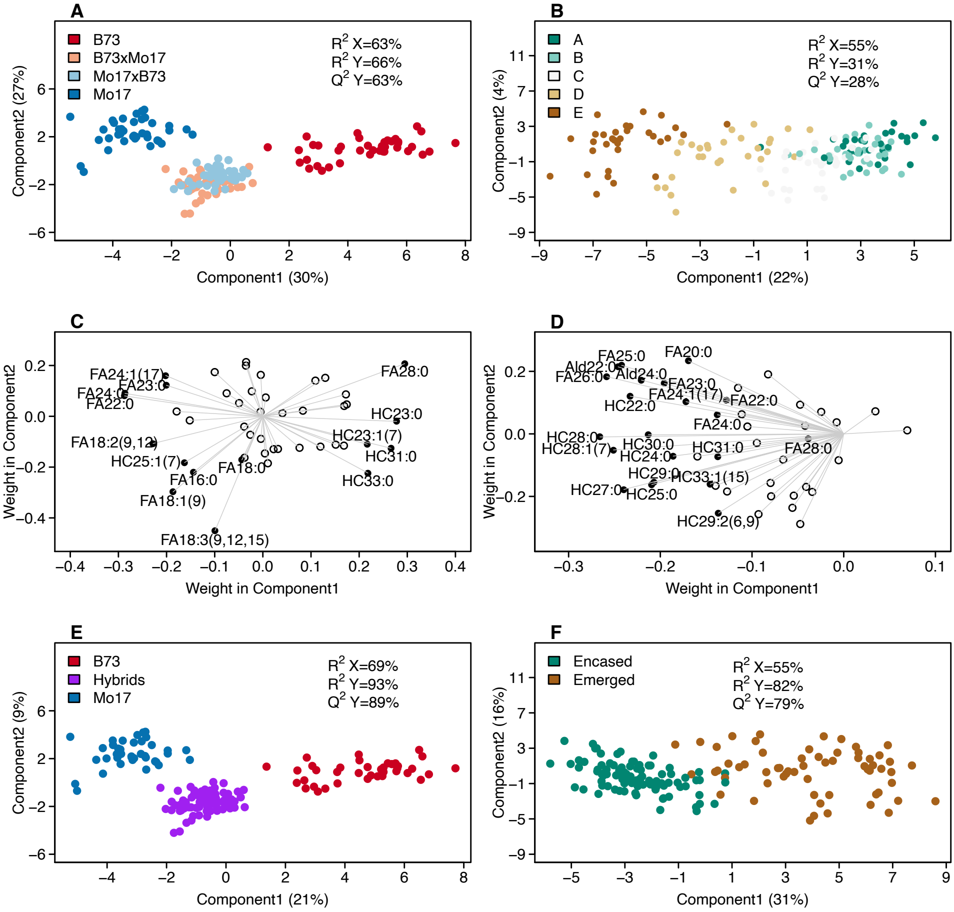

Supplemental Figure S3. Clustering of silk samples by partial least squares discriminant analysis (PLS-DA) based on different genotypes and different silk sections. PLS-DA score plots and corresponding weight plots based on different genotypes (**A, C,** and **E**) and silk sections (**B, D,** and **F**). The percent covariance between the response and predictor variables explained by each PLS component, R^2X, R^2Y, and Q^2Y for these PLS-DA models are reported in panels A and B. **C-D**, Weight plots, derived from the PLS-DA models presented in **A** and **B**, display the influence of each cuticular wax metabolite on the discrimination of different genotypes or silk sections. Closed circle symbols identify the signature metabolites selected with variable-importance-in-projection (VIP) scores >1. The 30 signature cuticular wax metabolites contribute to 41% and 24% for the R^2Y of the PLS-DA models that discriminate genotypes and silk-sections, respectively. **E-F**, revised PLS-DA models that discriminate the hybrids from the parental lines B73 and Mo17 (E), or discriminate the silk sections based on the encasement status (F). Abbreviations: Alc, alcohol; Ald, aldehyde; FA, fatty acid; HC, hydrocarbon.

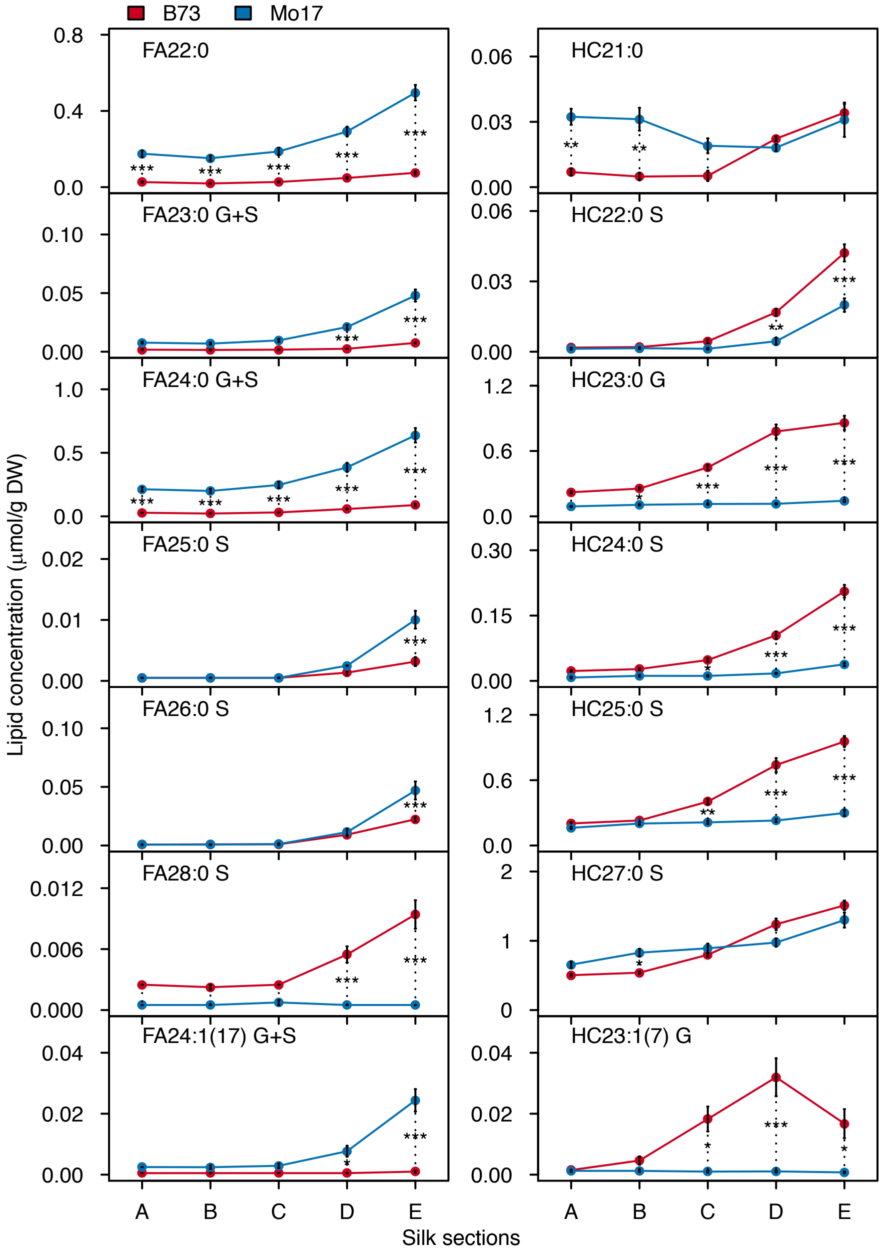

Supplemental Figure S4. The spatio-temporal accumulation patterns of the metabolically related hydrocarbon (*$HC_{2n\text{-}1:0}$*) and VLCFA (*$FA_{2n:0}$*) pairs in inbreds B73 and Mo17. These metabolites that were selected by PLS-DA as contributing to the discrimination of either genotype (G), silk section (S) or both (G+S) are identified. Data-points are the average ± standard error from 7-8 biological replicates. The genotype difference for each metabolite per silk section is represented by the length of the dotted lines. Asterisks denote statistical differences between genotypes at $p < 0.05$, according to Tukey's Honestly Significant Difference test (***, $p < 0.0001$; **, $p < 0.001$; *, $p < 0.05$). Abbreviations: DW, dry weight; FA, fatty acid; HC, hydrocarbon; VLCFA, very long-chain fatty acid.

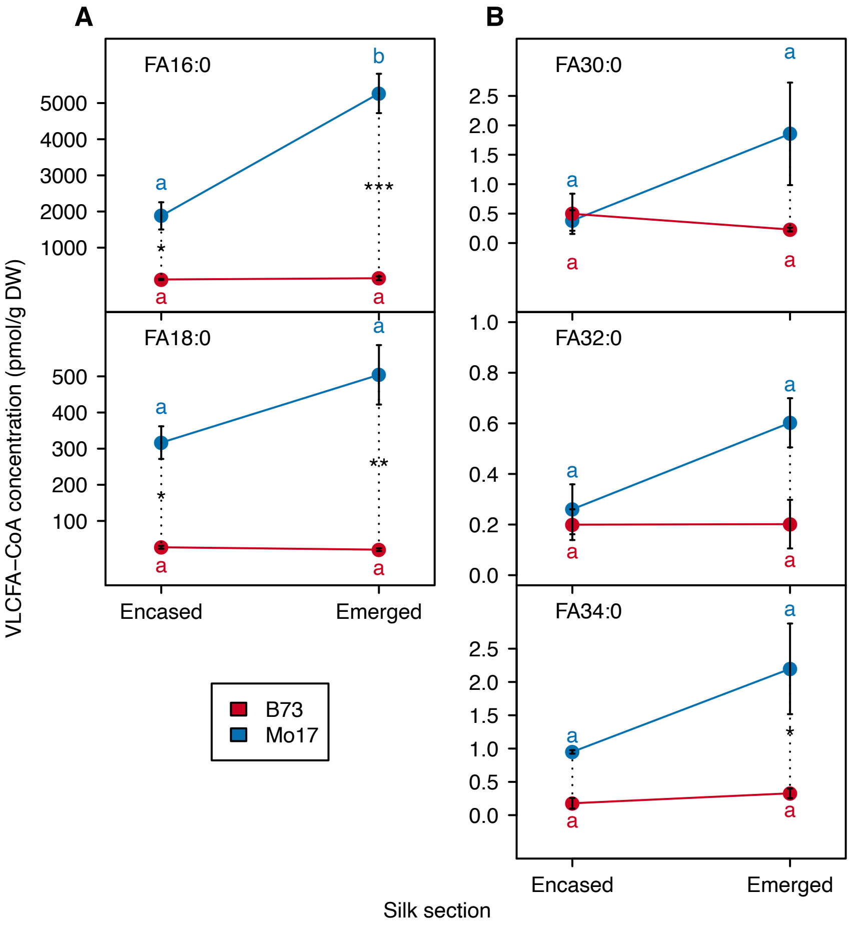

Supplemental Figure S5. Accumulation of fatty acyl-CoAs in the encased and the emerged sections of maize silks from inbreds B73 and Mo17. The concentrations of fatty acyl-CoAs with 16 or 18 carbons are presented in (**A**), and the concentrations of very long-chain fatty acyl-CoAs (VLCFA-CoAs) with 30 – 34 carbons are presented in (**B**). For each genotype, different letters associated with data points indicate statistical differences among silk sections (p <0.05; Tukey's Honestly Significant Difference test). The genotype difference for each metabolite per silk section is represented by the length of the dotted lines. Asterisks denote statistical differences between genotypes at a specific position along the silk length (***, p <0.0001; **, p <0.001; *, p <0.05; Tukey's Honestly Significant Different test). Averages ± standard errors from three biological replicates are reported for each fatty acyl-CoA. Abbreviations: DW, dry weight; FA, fatty acid.

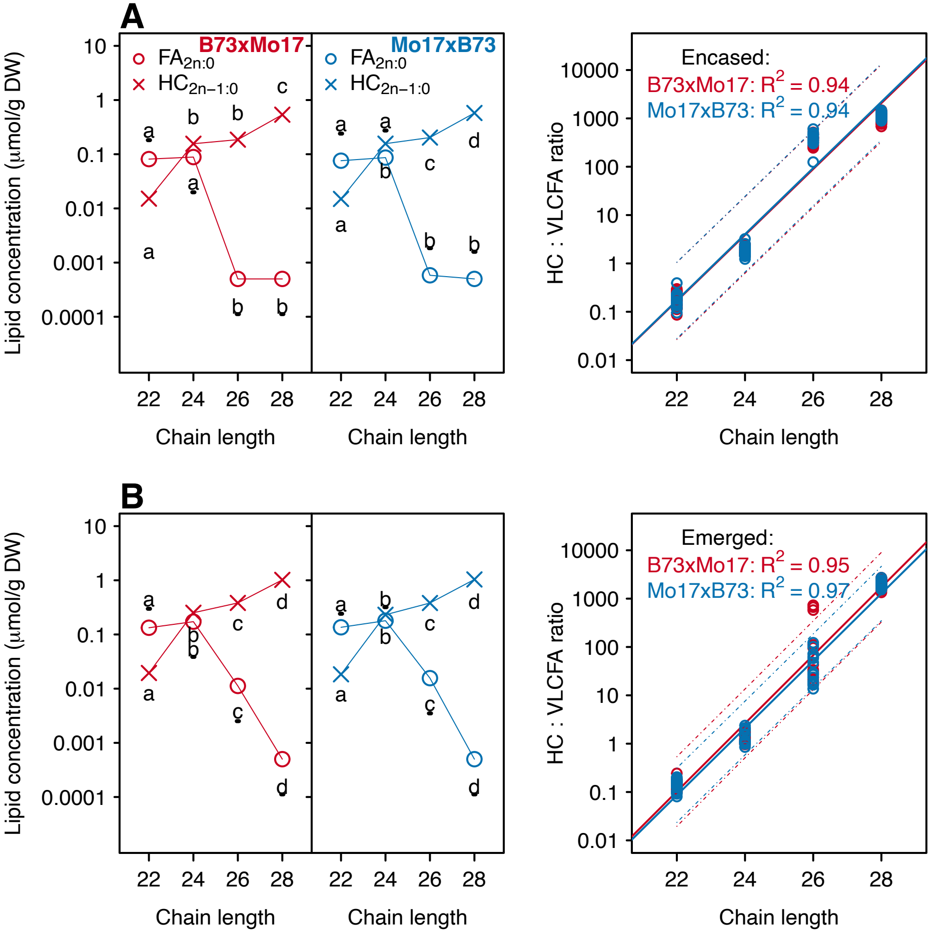

Supplemental Figure S6. Accumulation of cuticular hydrocarbon products and the metabolically related cuticular VLCFAs, and the regression of the hydrocarbon:VLCFA ratios on chain length for hybrids, B73×Mo17 and Mo17×B73. Concentrations (log-scaled) of cuticular hydrocarbons ($HC_{2n-1:0}$) and VLCFAs ($FA_{2n:0}$) were analyzed from husk-encased silks (**A**) and from silks that had emerged from the husks (**B**) of the hybrid B73×Mo17 (red data points) and Mo17×B73 (blue data points). Averages ± standard errors from seven to eight biological replicates are reported for the metabolite concentrations. Different letters associated with data points from the same metabolite class indicate a statistically significant difference between alkyl chain lengths ($p < 0.05$; Tukey's Honestly Significant Difference test); letters associated with VLCFAs are underlined. In the regression, the prediction intervals for the regression models are indicated by the dashed lines for each inbred. Abbreviation: DW, dry weight; FA, fatty acid; HC, hydrocarbon; VLCFA, very long-chain fatty acid.

Supplemental Statistical Methods

1. Calculation and comparison of Frobenius norms for correlation matrices

Comparison of correlation matrices among genotypes was performed according to Choi and Kendziorski (2009) with the modifications shown below. For each genotype, a value related to the Frobenius norm was calculated to evaluate the correlation level among metabolites within the cuticular lipid metabolome,

$$d_{G1} = \sqrt{\sum_{i<j} m_{ij}^2 / n(n-1)}$$

wherein n was the number of metabolites in the SL metabolome and $\{m_{ij}\}$, $1 \le i, j \le n$, represented the correlation matrix for genotype G_1, wherein m_{ij} was the correlation between metabolite i and j. The Frobenius norms for two genotypes (e.g., d_{G_1} and d_{G_2}) were compared by pairwise assessment of $d_{G_1G_2}\left(d_{G_1G_2} = d_{G_1} - d_{G_2}\right)$ under the hypothesis:

H_0(null hypothesis): $d_{G_1G_2} = 0$

H_1(alternative hypothesis): $d_{G_1G_2} \neq 0$

To test the null hypothesis, random simulations of genotypes $\boldsymbol{G_1}$ and $\boldsymbol{G_2}$ were generated by permuting the samples across two genotypes such that each simulated genotype contained data from both $\boldsymbol{G_1}$ and $\boldsymbol{G_2}$, and the resulting $\boldsymbol{d_{G_1G_2}}$was from a random population ($\boldsymbol{d^R_{G_1G_2}}$).

The p-value was equal to: $\dfrac{\mathbf{1}+\sum_i^B I(\left|d^{R,i}_{G1G2}\right| \geq |d_{G1G2}|)}{\boldsymbol{B}+\mathbf{1}}$

wherein $\boldsymbol{B}$ represents the number of permutations for $\boldsymbol{d}^{\boldsymbol{R}}_{\boldsymbol{G_1G_2}}$, and $\boldsymbol{d}^{\boldsymbol{R,i}}_{\boldsymbol{G_1G_2}}$ is the computed value of the genotype difference in the $\boldsymbol{i}$th permutation. H_0 was rejected when fewer than 5% of $\boldsymbol{d}^{\boldsymbol{R}}_{\boldsymbol{G_1G_2}}$ were smaller (when $\boldsymbol{d_{G_1G_2} < 0}$) or greater (when $\boldsymbol{d_{G_1G_2} > 0}$) than the experimentally measured $\boldsymbol{d_{G_1G_2}}$ (i.e. p-values < 0.05). The p-values were adjusted to control the false discovery rate ($\boldsymbol{\alpha}$ < 0.05) (Benjamini and Hochberg, 1995).

2. Determination of metabolite correlation networks

The correlation matrices were used to construct metabolite correlation networks using the R/WGCNA package (Langfelder and Horvath, 2008). First, the adjacency matrix that measures the similarity between two metabolites was calculated by soft thresholding a correlation matrix (Langfelder and Horvath, 2008) with a power value of six as suggested by Horvath and Langfelder (2011) and Dileo et al. (2011). Next, the topological overlap matrix, wherein each entry represents the number of metabolites that are connected to both "row" metabolite and "column" metabolite, was calculated based on the adjacency matrix. Finally, hierarchical clustering was applied by the hclust() function with the metabolite distance matrix calculated from the topological overlap matrix (Horvath and Langfelder, 2011). The metabolite clusters were determined by a consensus approach wherein four independent R functions were applied to prune the hierarchical trees: pam(), cutreeStatic(), and two cutreeDynamic()s with the "methods" argument specified as either "tree" or "hybrid".

3. Bayesian regression analysis for the dynamics of hydrocarbon:VLCFA ratios across precursor chain lengths

The hydrocarbon:VLCFA ratios ($^{HC_{n-1}}/_{FA_n}$) derived from the concentrations of cuticular hydrocarbons (HC_{n-1}) and cuticular VLCFAs (FA_n), were compared across precursor acyl

chain length n ($\in \{22, 24, 26, 28\}$) through Bayesian regression models. Two sets of Bayesian model comparisons have been performed using different combinations of metabolome data: Set 1) cuticular lipids (Fig. 1) and cellular VLCFA (free VLCFAs and GlcCer-associated VLCFAs within the silk; Supplemental Table S8) datasets from growing year 2015 to interrogate the association between the cuticular hydrocarbon:VLCFA ratios and cellular VLCFA concentrations (i.e., VLCFA model selection); and Set 2) cuticular lipid datasets from growing years 2014 and 2015, to interrogate the impact of growing year (i.e., Macroenvironment model selection). In all Bayesian regression models, the hydrocarbon:VLCFA ratio was the response variable and the acyl chain length was the fixed explanatory variable, with additional variables being included depending on the purpose of the model (e.g. genotype, husk-encasement status, free VLCFA concentrations, GlcCer-associated VLCFA concentrations). Bayesian model comparison was performed using the R package "BayesFactor" (Morey and Rouder, 2015).

Test 1: Bayesian regression analysis to test the impact of cellular VLCFA concentrations on the cuticular hydrocarbon:VLCFA ratio

Due to the differences in the number of replicates profiled for extracellular cuticular lipids *versus* total cellular lipids (i.e., 12-14 replicates *vs*. 3-4 replicates), VLCFA model selection has been performed on the 135 silk samples (out of 507) that were complete cases in which cuticular lipids, free VLCFAs and GlcCer-associated VLCFAs were profiled. Within the VLCFA model selection analyses, a baseline model was constructed that used VLCFA chain length, genotype, silk encasement status and the associated interactions as the predictors. Three VLCFA models that were modified from the baseline model were considered, each of which incorporated a class of cellular VLCFAs: VLCFA model 1) free VLCFAs (*ffa*) only; VLCFA model 2) GlcCer-

associated VLCFAs (*gfa*), only; VLCFA model 3) both free and GlcCer-associated VLCFAs (*ffa* and *gfa*).

For each of our three VLCFA models, we first measured the relative contributions of each of the interaction terms via Bayesian model comparisons. First, a full model was constructed by assuming that the hydrocarbon:VLCFA ratio y_{ngkvl}, in biological replicate l of chain length n, genotype g ($g \in$ {B73, Mo17}), encasement state k ($k \in$ {emerged, husk-encased}), and with the cellular VLCFA f ($f \in$ {*ffa*, *lfa*, *ffa and lfa*}), was generated through a normal regression model:

$$y_{nkgf} \overset{iid}{\sim} N(X\beta, \sigma^2) \text{ (\textit{iid} for independently and identically distributed)}$$

where X included the intercept, the factors and their interactions, i.e. $X = 1, n, g, k, f, g \times n, g \times k, k \times n, g \times k \times n, g \times f, k \times f, f \times n, g \times f \times n, k \times f \times n, g \times k \times f, g \times k \times f \times n$, and β was the vector of coefficients for each explanatory variable in X. The selection of priors for β and σ was detailed in Morey and Rouder (2015). The reduced models were constructed by iteratively removing one interaction term from the full model and calculating the likelihood ratio between this reduced model (denominator) and the full model (numerator). The resulting Bayes factors were then used to refine the full models by sequentially removing the interaction terms from the full model according to both the degree of the interaction (i.e., the number of factors involved) and the corresponding Bayes factor for each term, such that interactions with a higher degree and a Bayes factor > 3.2 were removed first and compared to the full model. This process optimized the three VLCFA models that incorporated *ffa*, *gfa*, or *ffa* and *gfa.*

Finally, using Bayesian model comparison, each of these optimized models (numerator) was compared to the baseline model (denominator), which did not incorporate the cellular VLCFAs. Optimized models with a Bayes factor >3.2 were considered to outperform baseline model.

Test 2: Bayesian regression analysis to interrogate the impacts of genetic background, husk encasement status, and growing year on the hydrocarbon:VLCFA ratio

Bayesian regression modeling was conducted on the hydrocarbon:VLCFA ratios from our multi-year dataset (i.e., Macroenvironment model selection), derived from cuticular lipid profiles from silks of two different encasement states ($k \in \{0, 1\}$) in two consecutive years ($v \in \{2014, 2015\}$), and across four genotypes ($g \in$ {B73, Mo17, B73xMo17, Mo17xB73}). In both 2014 and 2015, hydrocarbon:VLCFA ratios were calculated at the observed acyl chain lengths, $n \in \{22, 24, 26, 28\}$.

A baseline model was constructed by assuming that the hydrocarbon:VLCFA ratio y_{ngkvl} in biological replicate l of chain length n, genotype g, husk-encasement state k, and collected in year v, was generated through a normal regression model:

$$y_{nkgvl} \overset{\text{iid}}{\sim} N(X\beta, \sigma^2)$$

Where X included the intercept, and the factors to be examined as well as the interaction among these factors, i.e., $X = 1, g, k, v, n, g \times k, g \times n, k \times n, g \times k \times n,\ g \times v, k \times v, v \times n,$ $g \times k \times v, g \times v \times n,\ k \times v \times n, g \times k \times v \times n$, and β was the vector of coefficients for each explanatory variable in X. The selection of priors for β and σ was detailed in Morey and Rouder (2015). The Macroenvironment models were constructed by extracting one term each from the baseline model. As was performed for VLCFA model selection, Bayes factors that compared the

likelihood for the initial (numerator) and the modified (denominator) models were calculated by the R package "BayesFactor" (Morey and Rouder, 2018) to rank the impacts of these factors and their interactions on hydrocarbon:VLCFA ratios.

Literature Cited

Benjamini Y, Hochberg Y (1995) Controlling the false discovery rate: a practical and powerful approach to multiple testing. J R Stat Soc Ser B **57**: 289–300

Choi Y, Kendziorski C (2009) Statistical methods for gene set co-expression analysis. Bioinformatics **25**: 2780–2786

DiLeo M V., Strahan GD, den Bakker M, Hoekenga OA (2011) Weighted correlation network analysis (wgcna) applied to the tomato fruit metabolome. PLoS One **6**: e26683

Horvath S, Langfelder P (2011) Tutorial for the WGCNA package for R: **III. Using**: 5. Construction of a weighted gene co-expression n

Langfelder P, Horvath S (2008) WGCNA: an R package for weighted correlation network analysis. BMC Bioinformatics **9**: 559

Morey R, Rouder J (2018) BayesFactor: computation of bayes factors for common designs. R Package. version 0.9.12-4.2. https://CRAN.R-project.org/package=BayesFactor
